# Revealing the Host-Dependent Nature of an Engineered Genetic Inverter in Concordance with Physiology

**DOI:** 10.34133/bdr.0016

**Published:** 2023-08-16

**Authors:** Dennis Tin Chat Chan, Geoff S. Baldwin, Hans C. Bernstein

**Affiliations:** ^1^ Faculty of Biosciences, Fisheries and Economics, UiT, The Arctic University of Norway, 9019 Tromsø, Norway.; ^2^ Department of Life Sciences, Imperial College London, South Kensington, London SW7 2AZ, UK.; ^3^ Imperial College Centre for Synthetic Biology, Imperial College London, South Kensington, London SW7 2AZ, UK.; ^4^ The Arctic Centre for Sustainable Energy, UiT, The Arctic University of Norway, 9019 Tromsø, Norway.

## Abstract

Broad-host-range synthetic biology is an emerging frontier that aims to expand our current engineerable domain of microbial hosts for biodesign applications. As more novel species are brought to “model status,” synthetic biologists are discovering that identically engineered genetic circuits can exhibit different performances depending on the organism it operates within, an observation referred to as the “chassis effect.” It remains a major challenge to uncover which genome-encoded and biological determinants will underpin chassis effects that govern the performance of engineered genetic devices. In this study, we compared model and novel bacterial hosts to ask whether phylogenomic relatedness or similarity in host physiology is a better predictor of genetic circuit performance. This was accomplished using a comparative framework based on multivariate statistical approaches to systematically demonstrate the chassis effect and characterize the performance dynamics of a genetic inverter circuit operating within 6 Gammaproteobacteria. Our results solidify the notion that genetic devices are strongly impacted by the host context. Furthermore, we formally determined that hosts exhibiting more similar metrics of growth and molecular physiology also exhibit more similar performance of the genetic inverter, indicating that specific bacterial physiology underpins measurable chassis effects. The result of this study contributes to the field of broad-host-range synthetic biology by lending increased predictive power to the implementation of genetic devices in less-established microbial hosts.

## Introduction

While the collection of modular genetic parts (e.g., promoters and reporter proteins) has grown rapidly over the years, the number of genetically tractable “domesticated” microbial hosts, or chassis, has remained comparatively small. Indeed, despite a populous collection of bacteria readily available in culture collections [1], contemporary biodesign efforts still preferentially employ the same handful of model organisms (e.g., *Escherichia coli*, *Saccharomyces cerevisiae*, *Bacillus subtilis*, and *Pseudomonas putida*). While these model species are easy to work with, they do not always serve as the most optimal chassis for the intended objectives and could instead limit the potential of synthetic biology applications [2]. Broad-host-range (BHR) synthetic biology is an emerging field seeking to expand our engineerable domain beyond that of traditional model organisms and, in doing so, allows us to take advantage of the rich phenotypic diversity of naturally evolved microorganisms [3–5] to construct more sophisticated bespoke systems.

The number of studies promoting novel microbes for synthetic biology applications is increasing [6–12], demonstrating that the field of BHR synthetic biology has gained considerable traction. As synthetic biologists continue to explore the chassis design space, we (re-)discover that genetic circuits do not always maintain similar functional fidelity across hosts. Previous studies have shown that the same genetic circuit can exhibit significantly different behavior depending on the host environment it is operating within, an observation termed the “chassis effect” [13–16]. The chassis effect may hinder the accurate prediction of function from genetic composition alone [17], which can be disarming and lead to costly repetitions of the design–build–test cycle. The chassis effect can also render any optimizations of a circuit done in the context of a “design” host (typically a cloning-optimized strain) obsolete once transformed into the cellular environment of the destination host. This often discourages the use of nonmodel organisms. On the other hand, previous literature has demonstrated how the chassis effect can be exploited to expand the functionality and properties of circuits [15,16]. In this perspective, the host is viewed as a part that can be used to tune circuit function. The chassis effect can therefore act not only as an obstacle but also as an opportunity. However, a predictive understanding of which specific biological properties underpin observable chassis effects is lacking, representing a major knowledge gap that has been left unanswered partly due to default use of model organisms. Filling this knowledge gap will not only help mitigate the degree of uncertainty caused by the chassis effect but also provide more predictive power to BHR synthetic biology applications and contribute to broadening the design space available for biodesign applications.

Studies in the field of biosynthetic gene cluster expression [18] and microbial community engineering [19] have shown that similarities in phylogeny and genotypic profiles can predict metabolic phenotype, suggesting that genome relatedness could be a potential predictor of genetic circuit performance. On the other hand, the functional phenotype of genetic devices has been shown to be coupled to physiological metrics such as growth rate [20,21], gene copy number [22,23], codon usage bias [24,25], and growth burden [26,27] in a number of studies. However, these studies have only considered a few or single physiology metrics as explanatory variables within a single model chassis. Here, we detail a comprehensive study that takes account a multitude of explanatory variables within a comparative framework that includes model and nonmodel bacterial chassis. We systematically demonstrate the chassis effect by characterizing the performance dynamics of a genetic inverter circuit, as an example of an engineered genetic circuit, within 6 different Gammaproteobacteria species. As our guiding research question, we ask whether phylogenomic relatedness or similarity in assorted host physiology metrics is a better predictor of genetic inverter circuit performance. Due to the more extensive documentation on the coupling of host physiology and gene expression, we hypothesized that variations in physiology between hosts will more robustly predict variations in performance of a genetic device, in comparison to phylogeny.

## Methods

Experimental design

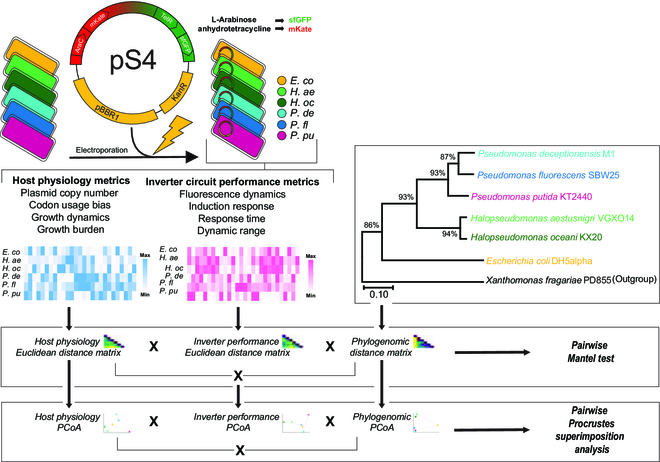

**Experimental design figure.** This study was guided by the following research question: Is phylogenomic relatedness or host physiology a better predictor of circuit performance? To answer this, an l-arabinose (Ara)- and anhydrotetracycline (aTc)-inducible inverter circuit (plasmid pS4) was introduced into 6 Gammaproteobacteria species. Inverter performance and host physiology of hosts was quantified under comparable standardized conditions, and phylogenomic relationship of the species was resolved. (Dis-)Similarity between hosts in terms of host physiology and inverter performance was determined, resulting in 2 Euclidean distance matrices and a phylogenomic distance matrix. Significant correlation between distance matrices was tested in pairwise fashion using the Mantel test. For further analysis, distance matrices were projected onto ordinate space by principal coordinate analysis (PCoA) and Procrustes Superimposition analysis was performed.

## Strains and plasmids

Wild-type (WT) species were purchased from the German Collection of Microorganisms and Cell Cultures (DSMZ), except for *Pseudomonas fluorescens* SBW25, which was donated by R. Wilton from the Argonne National Laboratory (Illinois, USA). Bacterial strains and plasmids used in this study are summarized in Table S2. Primers used are listed in Table S3. All cloning work was performed in *E. coli* DH5α, which was made chemically competent and transformed following the Inoue method [28].

## Media and culturing conditions

All cells were cultured in lysogeny broth (LB) at 30 °C unless specified otherwise. Inverter-carrying strains were cultivated with 100 μg/ml kanamycin, while WT counterparts were grown without kanamycin. Overnight cultures were prepared by inoculation with single colonies from streaked plate and cultured with shaking. Ninety-six-well plate cultivation was done in black and flat clear-bottom plates (Thermo Fisher Scientific, 165305). Medium (199 μl) was inoculated with 1 μl of culture and sealed with a Breathe-Easy film (Sigma-Aldrich, Z380059). OD_600_, sfGFP (485/515, gain = 75), and mKate (585/615, gain = 125) fluorescence was measured continuously up to 42 h using a Synergy H1 plate reader (Agilent Biotek, serial number 21031715) with continuous linear shaking (1096 cpm, 1 mm). l-Arabinose stock (1 M) was prepared by dissolving l-arabinose (VWR, A11921) in water, filter sterilized and stored at 4 °C. aTc stock (1 mg/ml) was prepared by dissolving anhydrotetracycline hydrochloride (VWR, CAYM10009542) in 70% ethanol and stored at −20 °C away from light.

## BASIC assembly

Plasmid pS4 was assembled with biopart assembly standard for idempotent cloning (BASIC) [29,30]. For complete protocol, see Storch et al. [29]. DNA parts used in this study are listed in Table S4. Briefly, BASIC assembly is a “one-pot” DNA assembly method for joining DNA parts, following their integration into the BASIC standard. BsaI-HFv2 restriction enzyme and T4 DNA ligase was purchased from New England Biolabs (R3733 and M0202L, respectively). Mag-Bind TotalPure NGS (Omega Bio-Tek, M1378-01) was used to purify restriction–ligation reactions as per the manufacturer’s instructions. BASIC linkers were purchased from Biolegio (BASIC Linkers Pro-Plate Set, BBT-18500). pSEVA231 plasmid was donated by the SEVA repository and integrated into the BASIC format with B_SEVA_F and B_SEVA_R primers using the KAPA HiFi HotStart ReadyMix PCR Kit with the following program: initial denaturation at 95 °C for 180 s, followed by 30 cycles of the following: 98 °C for 20 s, 58 °C for 15 s, 72 °C for 150 s, and final extension step at 72 °C for 150 s. *AraC*, *TetR*, *sfGFP*, and *mKate* parts were ordered from Twist Biosciences, with the BASIC *i*P and *i*S sequences integrated at the 5′-end and 3′-end, respectively.

## Preparation of electrocompetent cells and electroporation

All incubation steps were done at 30 °C unless specified. Overnight culture (5 ml) was diluted by half with fresh LB medium and incubated for 1 h. For *Halopseudomonas oceani* KX20 and *Halopseudomonas aestusnigri* VGXO14, 5 ml of culture was harvested per transformation. For all other, 1.5 ml was harvested per transformation. Harvesting was done by centrifugation at 4,000 rpm at room temperature. Supernatant was discarded and cells were resuspended in sucrose electroporation buffer (300 mM sucrose, 1 mM MgCl, pH 7.2). The washing step was repeated for a total of 2 washes. Cells were finally resuspended in 80 μl of electroporation buffer and incubated with 50 to 100 ng of plasmid at room temperature for 15 min. Cells were transferred to a 1-mm gap electroporation cuvette (VWR, 732-1135) and electroporated using the ECM 399 Exponential Decay Wave Electroporation System (BTX, 45-0000) at 1,250 V with high voltage setting (150 resistance and 36 capacitance). LB medium (750 μl) was immediately added to electroporated cells and subsequently transferred to 5 ml of LB for recovery by incubation with shaking for 2 h. After recovery, cells were harvested and streaked on kanamycin LB agar plates. Colonies would appear after 1 or 2 overnight incubations. Colonies were verified by colony PCR with LMP-F and LMS-R primers.

## Induction assay

Ninety-six-well plates with kanamycin supplemented LB medium and various concentrations of Ara and aTc were inoculated with overnight culture grown in the absence of inducer. Normalized steady-state fluorescence intensity was quantified by averaging fluorescence over a time window of 6 to 12 h at late phase (y_Fss_) and plotted against induction concentration to yield induction curves. Hill coefficient (*n*), activation coefficient (*K*), maximum steady-state fluorescence output (β), and basal fluorescence output at 0 inducer concentration (*y*_0_) were estimated by fitting the Hill equation (Eq. 1) to induction curves using nonlinear least-square regression with the *nls* function from the *stats* base R package.

$$y_{\mathrm{Fss}}=\left(\beta x^{n}/\left(K^{n}+x^{n}\right)\right)+y_{0}$$ (1)

where *x* is Ara (mM) or aTc (ng/ml) inducer concentration.

## Toggle and growth assay

Based on the induction assay, inducer concentrations of 20 mM Ara and 10 ng/ml aTc were chosen to induce all inverter-carrying species. Overnight culture grown in the absence of inducer was used to inoculate the medium in 96-well plates supplemented with Ara, aTc, and no inducer (NI) condition. OD_600_, sfGFP, and mKate fluorescence was continuously measured. To invert states, cells were harvested by centrifugation at 4,000 rpm for 20 min at room temperature and supernatant removed before resuspending in equal volume LB medium; this washing step was repeated for a total of 2 washes. Washed resuspended cells (1 μl) were inoculated to 199 μl of fresh medium supplemented with the opposite respective inducer. Metrics Δμ_NI_, Δμ_Ara_, and Δμ_aTc_ were calculated using Eqs. 2, 3, and 4, respectively.

$$∆\mu_{\mathrm{NI}}=((\mu_{\mathrm{WT}\_\mathrm{NI}}-\mu_{pS4\_\mathrm{NI}})/\mu_{\mathrm{WT}\_\mathrm{NI}}\;)100$$ (2)

$$∆\mu_{\mathrm{Ara}}=((\mu_{\mathrm{pS}4\_\mathrm{NI}}-\mu_{\mathrm{pS}4_{\mathrm{Ara}}})/\mu_{\mathrm{pS}4_{\mathrm{NI}}})\;100$$ (3)

$$∆\mu_{\mathrm{aTc}}=((\mu_{\mathrm{pS}4\_\mathrm{NI}}-\mu_{\mathrm{pS}4_{\mathrm{aTc}}})/\mu_{\mathrm{pS}4_{\mathrm{NI}}})\;100$$ (4)

μ_WT_NI_ is the specific growth rate of the WT strain without inducer. μ_pS4_NI_, μ_pS4_Ara_ and μ_pS4_aTc_ are the specific growth rates of the pS4-carrying counterpart strain in the absence of inducer, in the presence of 20 mM Ara, and in the presence of 10 ng/ml aTc, respectively. Maximum rates of OD_600_ and fluorescence curves were estimated based on a rolling regression method using the *all_easylinear* function from the *growthrates* (v.0.8.4, https://CRAN.R-project.org/package=growthrates) R package. Lag times and curve plateaus of OD_600_ and fluorescence curves were determined using the *all_growthmodels* function, fitting the Gompertz growth model [31] with additional lag (lambda) parameter.

## Flow cytometry and hysteresis experiment

Ara, aTc, and noninduced cells were harvested during late exponential phase and fixed with formaldehyde to a final concentration of 1.5% and standardized to OD_600_ = 0.2. Flow cytometry was performed with the LSRFortessa Cell Analyzer (BD Sciences) equipped with an HTS autosampler (BD Sciences). sfGFP was measured using 488-nm laser and 530/30-nm detector, and mKate was measured using 561-nm laser and 610/20-nm detector. Voltages for forward scatter and side scatter sfGFP and mKate detection was set to 680, 380, 440, and 550, respectively. Twenty thousand events were measured per sample with side scatter threshold set to 40,000 to filter out background debris. Hysteresis experiment was based on Zhang et al. [20]. Single colonies were inoculated to fresh LB medium and allowed to grow overnight. Cells were diluted 1:100 to fresh medium supplemented with 80 mM l-arabinose, 150 ng/ml aTc, NI-condition and grown to stationary phase. Cells were washed twice before being diluted 1:100 to medium containing increasing concentration of the inducer, and fluorescence was measured continuously on a plate reader.

## Plasmid copy number determination

Plasmid Copy Number (PCN) per chromosome was determined through quantitative PCR (qPCR) method based on [32] using the single-copy *rpoD* gene and the *bla* gene in pS4 as amplification targets. Primers for qPCR were designed with BatchPrimer3 [33] with the following criteria: product size = 100 to 125 base pairs (bp), primer size = 20 to 24 bp, primer Tm = 58 to 62 °C, primer GC % = 45 to 55%. Cells were harvested at stationary phase (18 to 24 h) by centrifugation at 4,500 rpm, 4 °C for 10 min and resuspended to OD_600_ = 1 in cold PBS buffer (1×, pH 7.3). Up to 1 ml of resuspended culture was incubated at 95 °C for 20 min to lyse the cells and immediately stored at −20 °C until use as template for qPCR. Simultaneously, resuspended culture was serially diluted to 10^−9^ and 100 μl of 10^−9^, 10^−8^, 10^−7^, and 10^−6^ dilutions was streaked on LB agar with kanamycin and incubated overnight at 30 °C for viable cell count. Lysed template samples were diluted to dynamic range of 10^2^ to 10^5^ bacteria per well. qPCR was performed in 20-μl reaction volumes containing 10 μl of KAPA SYBR FAST qPCR Master Mix (2X) (Sigma-Aldrich, KK4605), 200 nM final concentration of species-specific *rpoD* forward and reverse primers, and up to 3 μl of sample template in a fast optical 0.1-μl 96-well plate (Applied Biosystems, 4346906). Separate reactions were done for chromosomal and plasmid amplicons, each in 4 technical replicates. qPCR was performed using the 7500 Fast Real-Time PCR System (Applied Biosystems, 4351107) with the following cycling conditions: 1 min at 95 °C followed by 40 cycles of 3 s at 95 °C and 30 s at 60 °C. A melt-curve analysis step was added with the following program: 15 s at 95 °C, 1 min at 60 °C, followed by a 1% gradual increase in temperature to 95 °C and 15 s at 60 °C. Cycle threshold (*C*_t_) values were determined after automatic adjustment of the baseline and fluorescence thresholds in 7500 Software v2.3 (Applied Biosystems). Relative standard curves were constructed in R by plotting log value of the number of colony-forming units against *C*_t_ values. Slopes were determined from curves with 3 to 5 dilutions and on condition that *r*^2^ ≥ 0.99. Amplification efficiency (*E*) for plasmid and *rpoD* was calculated, which was used in turn to determine PCN using Eq. 6.

$$E=10^{-1/\text{slope}}$$ (5)

$$\text{PCN}={E\_c}^{\mathrm{Ct}\_c}/{E\_p}^{\mathrm{Ct}\_p}$$ (6)

E_c is the amplification efficiency for *rpoD* gene (chromosome), and E_p is the amplification efficiency for *bla* gene (plasmid).

## Phylogenomic and phylogenetic tree building

See Table S2 for genome accession numbers. GToTree [34] (v.1.7.07, https://github.com/AstrobioMike/GToTree) was run with default settings using the Gammaproteobacteria HMM set for 172 orthologous single-copy genes to create an amino acid multilocus sequence analysis (MLSA). Tree inference and distance matrix construction was done in MEGAX [35] using the neighbor-joining method with the Jones–Taylor–Thornton matrix-based model [36] with uniform rates. Ambiguous positions were removed for each sequence pair (pairwise deletion option), and standard error estimates were obtained with bootstrap procedure of 1000 replicates.

## Codon adaption index

To determine codon adaption index (CAI), the synonymous codon usage of genes encoding ribosomal proteins and ribosomal RNA genes was used as reference for each host, which are genes assumed to be highly expressed and thus under selection pressure to use synonymous codons adapted to the codon usage bias of the organism [37,38]. Sequences of ribosomal proteins and 16*S* and 23*S* ribosomal RNA were retrieved using *anvi-get-sequences-for-hmm-hits* function with the *Bacteria_71* HMM set in the anvi’o [39] (v.7.1, https://github.com/merenlab/anvio) environment. The CAI for *sfGFP*, *mKate*, *AraC*, and *TetR* genes were calculated using the standard genetic code with the CAI python package [40] (v.1.0.2, https://github.com/Benjamin-Lee/CodonAdaptationIndex). CAI is the geometric mean of the relative adaptiveness of each codon in a given sequence with the following implementation in the CAI python program:

$$\mathrm{CAI}={\left(\prod_{k=1}^{L}\mathrm{Wk}\right)}^{1/L}$$ (7)

*L* is the number of codons, and *Wk* is the relative adaptiveness of codon *k* and is calculated as follows:

$$\mathrm{Wk}=F_{\mathrm{ij}}/F_{\text{iMax}}$$ (8)

*F*_ij_ is the frequency of codon *j* for coding for amino acid *i* in a given sequence. *F*_iMax_ is the frequency of the most optimal codon for amino acid *i* in the given sequence, with the most optimal codon being the one with the highest frequency in the reference set of genes.

## Statistical analyses

Generation of Euclidean distance matrices (equal weights), Principal Coordinate Analysis (equal weights), Mantel test, and Procrustes Superimposition (PS) analysis was done using the Vegan [41] (v.2.6-4, https://CRAN.R-project.org/package=vegan) package in R. The Procrustean *M*^2^ statistic (scale and symmetric true) and Mantel *R* statistic (Pearson method) were tested for significance by a permutation approach (*n* = 719, maximum number of iterations). Briefly, observations in one matrix are randomly reordered while maintaining the covariance structure within the matrix and a test statistic is calculated and recorded enough times to obtain a sizeable null distribution. A *P* value for each statistic is then calculated, representing the probability of obtaining a statistic with a value equal to or more “extreme” of the experimental value.

## Results

### Comparative physiology and phylogeny between hosts

**Fig. 1.**
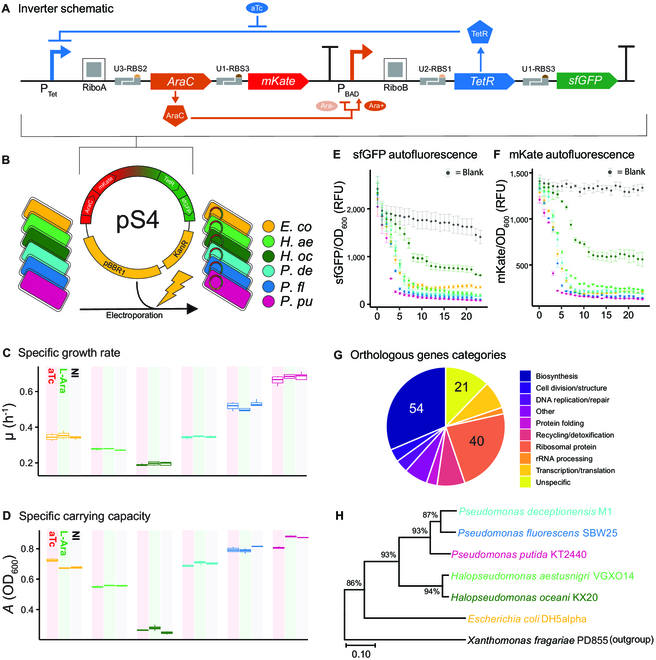
The broad-host-range pS4 plasmid harboring the inverter circuit was introduced into 6 distinct hosts from the Gammaproteobacteria class. (A) Schematic of Ara- and aTc-inducible inverter circuit. In the presence of aTc, the upstream cassette is up-regulated, leading to production of AraC transcription factor and mKate reporter protein. In the absence of Ara (Ara−), AraC acts as a repressor and binds to its cognate *P*_BAD_ promoter to down-regulate the downstream cassette. When bound to Ara (Ara+), AraC instead acts as an activator and up-regulates expression of sfGFP and TetR, creating a negative feedback loop of AraC expression. RiboA and RiboB are autocatalytic ribozyme insulators. U*n*-RBS*n* are BASIC linkers with a RBS in their adapter region. Number in U*n* indicates the BASIC linker family (1, 2, or 3). Number in RBS*n* indicates relative translational strength of the RBS, from 1 (weakest) to 3 (strongest). (B) Plasmid pS4 was transformed by electroporation into six bacterial Gammaproteobacteria hosts. Hosts are color-coded. *E. co*, *E. coli*; *H. ae*, *H. aestusnigri*; *H. oc*, *H. oceani*; *P. de*, *P. deceptionensis*; *P. fl*, *P. fluorescens*; *P. pu*, *P. putida*. (C) Specific growth rate (μ) and (D) carrying capacity (*A*) of WT strains in the presence of aTc (10 ng/ml) and Ara (20 mM) and no inducer (NI). Error bars show standard error of the mean (*n* = 3 biological replicates, with 4 technical replicates each). sfGFP (E) and mKate (F) autofluorescence normalized by OD_600_ over time by WT strains in the absence of inducer. Blank is autofluorescence of wells containing only LB medium. (G) The 172 single-copy genes in the Gammaproteobacteria hidden Markov model set from GToTree used for phylogeny inference grouped by functional annotation. Numbers of genes in the largest grouped are indicated. (H) Phylogenomic tree inferred from multi-locus sequence alignment of concatenated gene hits. *Xanthomonas fragariae* PD855 was chosen as outgroup. Scale bar indicates the number of amino acid substitutions per site between 2 sequences. Tree was inferred using the neighbor-joining method in MEGAX. The percentage of replicate trees in which the associated taxa clustered together in bootstrap test (1,000 replicates) are shown next to the branches.

Measuring biological determinants of the chassis effect requires a standardizable, BHR device that is portable across multiple species. We therefore built a tractable experimental platform by transforming six Gammaproteobacteria species with a genetic inverter circuit cloned onto a pSEVA231-derived (kanamycin selection marker and pBBR1 origin of replication) vector (Fig. 1A and B) within the BASIC cloning environment [29,30], yielding plasmid pS4. The inverter consists of 2 inducible antagonistic expression cassettes reported by mKate and/or sfGFP fluorescence signals. This topology allows cells carrying the inverter to achieve 2 distinct ON states in the presence of either aTc or Ara input inducers. The inverter was successfully transformed into the following six species: *E. coli*, *H. aestusnigri*, *H. oceani*, *Pseudomonas deceptionensis* M1, *P. fluorescens*, and *P. putida*. Members of the *Pseudomonas* and *Halopseudomonas* genus were specifically chosen because of their known robustness and metabolic capabilities [42,43], making them attractive candidates for biotechnology applications and for comparing bacterial ecophysiology.

A preliminary growth characterization of WT strains in the absence and presence of Ara and aTc inducer was performed (Fig. 1C and D). Each species revealed distinct growth physiologies, allowing for quantitative comparison of the relative contribution that a host’s physiology plays on device performance. In the absence of any inducer, *P. putida* achieved the highest maximum specific growth rate (μ = 0.67 h^−1^ ± 0.01), while the 2 other *Pseudomonas* spp. exhibited lower growth rates compared to *P. putida,* but reached similarly high specific carrying capacities (*A*; Fig. 1C and D). Meanwhile, the 2 *Halopseudomonas* members showed approximately 2- to 3-fold lower μ compared to *P. putida*. Given the documented impact of growth rate on functional output of genetic devices, such as how rapid cell division results in dilution of gene expression machinery and expression products [20], the range of growth dynamics observed leads us to predict that a measurable chassis effect would be observed among our hosts. No appreciable difference in growth rate or carrying capacity was observed when the WT strains were grown in the presence of either inducer, suggesting negligible levels of toxicity. Furthermore, many *Pseudomonas* species are known to naturally fluoresce, which could mask the output of the inverter. At the excitation and emission wavelength settings used to detect sfGFP and mKate, none of the species had significant levels of autofluorescence (Fig. 1E and F), validating the use of the 2 fluorescent proteins chosen as reporters.

The relative performance of the inverter circuit is expected to be in part related to the genomic potential of the host. For example, genes encoding for the specific transcriptional and translational proteins used to express the genetic circuit’s machinery will ultimately underpin performance. We used a phylogenomic approach to ask if hosts more genomically related also share similar circuit performances. Orthologous single-copy genes fit for phylogeny inference in Gammaproteobacteria were identified in each host genome using a set of 172 hidden Markov models (Fig. 1G) from GToTree [34]. The set included genes encoding for ribosomal proteins, chaperones, and transcription and translation proteins, which all play some role in shaping the gene expression landscape of each host such as steady-state concentration of transcriptional/translational machinery or turnover rate and are therefore expected to influence device performance. The genomes with the lowest gene hits identified after filtering for redundancy and length belonged to *Xanthomonas fragariae* PD855 (outgroup) and *P. fluorescens*, both with 159 hits, while the other 5 genomes retained between 166 and 170 genes (Table S1). The phylogenomic tree clustered the hosts into distinct clades according to their genus (Fig. 1H), as expected. The growth characterization indicates that closely related hosts also shared similar growth physiologies. These results set the stage to comparatively investigate how the performance of the inverter circuit relates the relative contribution of species-specific physiology as compared to phylogenomic relatedness (i.e., more similarly evolved genomes) and to ask whether host physiology or phylogeny is a more robust predictor of differences in the performance of a genetic device between hosts.

### Quantifying the chassis effect

**Fig. 2.**
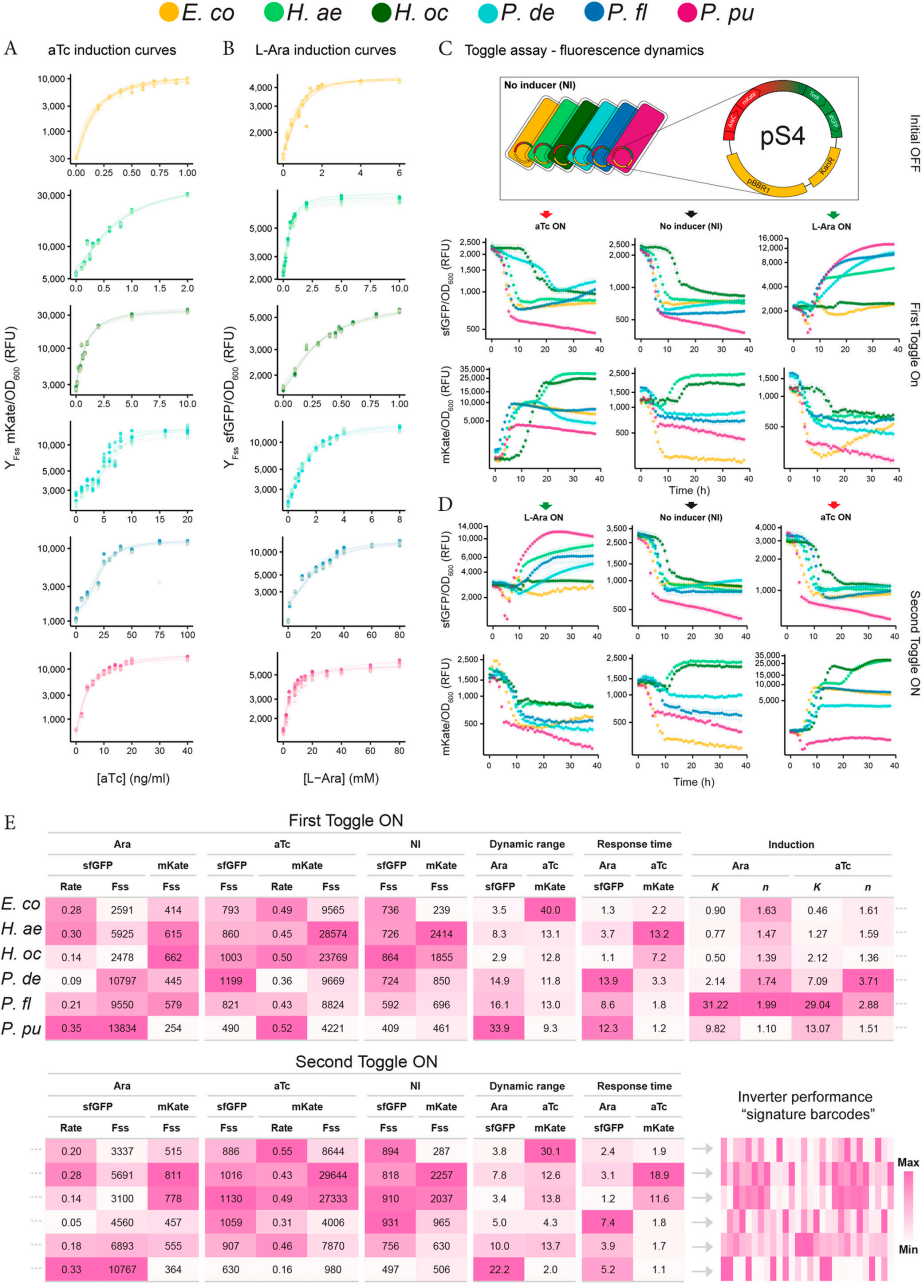
The chassis effect as observed through heterogeneity of performance metrics of the inverter circuit. (A) aTc and (B) Ara induction curves of pS4-carrying hosts. *Y* axis in log scale and parameter fitted Hill function are shown (*n* = 8). Hosts are color-coded according to the legend on the top of the figure. (C) Normalized sfGFP and mKate fluorescence of hosts toggled from initial OFF state (NI) to ON state (First Toggle ON) with 20 mM Ara (right, green arrow) and 10 ng/ml aTc (left, red arrow). (D) Cells were toggled by washing and diluted to medium with opposite inducer at the same concentration (Second Toggle ON). (E) Summary of performance metrics estimated from induction and toggle assay experiments for each host, with each column representing a metric. Gray dots denote continuation of table. Rate = maximum rate in unit of h^−1^, Fss = estimated fluorescence steady state at stationary phase of growth, Response time in units of hours, *K* = activation constant, *n* = Hill coefficient. Color scale denoting “Max” and “Min” value is relative for each column.

All hosts carrying the inverter circuit achieved uniform fluorescence distribution upon induction, as revealed by flow cytometry (Fig. S1). Induction kinetic and expression dynamic assays were performed to quantify inverter performance operating under the cellular context of each chassis. Significant differences in performance were observed between chassis, establishing a clear and quantifiable chassis effect (Fig. 2).

Induction kinetics of the inverter were resolved by fitting the Hill equation to Ara and aTc induction response curves to estimate the species-specific activation constants (*K*_Ara_ and *K*_aTc_) and Hill coefficients (*n*_Ara_ and *n*_aTc_) (Fig. 2A and B). *P. fluorescens* exhibited the largest inducible range for both inducers (*K*_Ara_ = 31.2 mM ± 2.1 and *K*_aTc_ = 29.0 ng/ml ± 1.1). Meanwhile, *E. coli* and both *Halopseudomonas* spp. demonstrated some of the lowest activation coefficients. The differences in activation constants between, and within, hosts represent how each host uniquely mediates the concentration and/or binding affinities of free intracellular inducer that is available to allosterically bind its cognate repressor. Both sigmoidal responses and damped “titratable responses” between hosts across induction states were observed, but all Hill coefficients were estimated to be close to or higher than 1. The Hill coefficients *n*_Ara_ and *n*_aTc_ did not differ drastically within hosts, the only exception being *P. deceptionensis*, which exhibited a more step-like response behavior to aTc (*n*_aTc_ = 3.71 ± 0.53) compared to Ara induction (*n*_Ara_ = 1.74 ± 0.17). Clearly, the choice of chassis can greatly impact the operational properties of the inverter, demonstrating the importance of considering the chassis during design stages.

To further quantify the chassis effect, fluorescence dynamics of hosts under the same induction condition were characterized through a toggling assay (Fig. 2C). Overnight cultures grown under NI conditions were diluted to medium containing Ara or aTc to toggle each respective promoter ON. Interestingly, when diluted to NI conditions, cells consistently assume a low expression state of both fluorescent proteins (Fig. 2C, middle). We refer to this stable low-expression state as the OFF state. To quantify host-specific fluorescence dynamics, maximum rate (Rate) and the fluorescence steady state at stationary phase of growth (Fss) was estimated from normalized sfGFP and mKate curves across induction states. We also determined two additional metrics “dynamic range” (DR) and “Response Time,” which we define here as the ratio between induced Fss and noninduced OFF state Fss (DR_sfGFP_ = Fss_Ara-sfGFP_/Fss_NI-sfGFP_ and DR_mKate_ = Fss_aTc-mKate_/Fss_NI-mKate_) and the time it takes to reach half of the Fss (subtracting for time during lag phase), respectively.

Under Ara induction, the three *Pseudomonas* spp. display the highest Fss_Ara-sfGFP_ values, which reflect the concentration of sfGFP attained in the population. *P. putida* attained the highest value (Fss_Ara-sfGFP_ = 13,833 RFU ± 937) and the highest dynamic range (DR_sfGFP_ = 33.85 ± 1.37) (Fig. 2C and E). However, the high sfGFP output level from these three chassis were also associated with long response times. *E. coli*, *H. aestusnigri*, and *H. oceani* had not only lower DR_sfGFP_ values but also shorter response times. Under aTc induction, the highest Fss_aTc-mKate_ values were reached by the two *Halopseudomonas* spp., again associated with long response times. *E. coli*, however, exhibited the highest DR_mKate_ value, with an induced mKate output level 40-fold that of the output level in the absence of inducer. Even in the presence of repressor or in the absence of activator, some basal level of expression was observed from an inducible promoter, referred to as leakage [44]. We identify two types of leakages in our inverter circuit: output in the absence of inducer (OFF state, Fig. 2C, middle) and output in the presence of the expression cassette’s antagonistic inducer (i.e., sfGFP in the presence of aTc and mKate in the presence of Ara). Under these conditions, the Fss at late phase was used to quantify leakage. OFF state cells exhibit relatively low expression, indicating similarly basal activity of the promoters among hosts. The exception was *H. aestusnigri* and *H. oceani*, which showed mKate Fss values about 10- and 7-fold higher than *E. coli*, suggesting that the *P*_Tet_ promoter is stronger when operating in the cellular context of the two *Halopseudomonas* chassis. We also observed up to 4-fold lower mKate leakage in the two *Halopseudomonas* spp. under Ara induction compared to the leakage in the absence of inducer, which we attribute to repression of the *P*_Tet_ promoter by expressed TetR. The wide range of metrics observed from different species cultivated and induced under the same conditions further reinforces the presence of the chassis effect affecting inverter performance.

Cells toggled to Ara/aTc-ON from initial OFF were washed and diluted to the opposite induction state (Fig. 2D, Second Toggle ON) to investigate whether cells experience appreciable hysteresis effect [20,45,46]. When toggled from aTc-ON to Ara-ON, the three *Pseudomonas* spp. consistently experienced a decrease in DR_sfGFP_ values, ranging from 0.78- to 0.42-fold change (Fig. 2E) compared to cells toggled to Ara-ON from the initial OFF state, although this was accompanied with a 0.54- to 0.43-fold change in response time as well. As cells were washed of inducer and diluted, this decrease in expression can be attributed to the cells past the induction state, suggesting a dependence on past induction states. When toggled from Ara-ON to aTc-ON, *P. putida* and *P. deceptionensis* showed a notable 0.37- and 0.21-fold change decrease in DR_mKate_, respectively. *H. aestusnigri* and *H. oceani* showed similar fluorescence dynamic metrics regardless of past induction state. This observation prompted an investigation on whether our inverter-carrying cells can retain memory of their past induction state. Inducer-saturated cultures were washed and diluted to NI and various inducer concentrations. *P. deceptionensis* and *P. putida*, and all other hosts to a lesser extent, exhibit a clear concentration range where the same aTc concentration elicits a different degree of fluorescence response between saturated and initial OFF cells (Fig. S2). Interestingly, when saturated with Ara, none of the hosts exhibited any appreciable retention of past induction state. These results indicate that, under our induction scheme, some hosts are more sensitive to past states of induction than others, another host-specific property.

Abstracting and comparing quantified performance metrics as “signature barcodes” reveal a clear heterogeneity in performance among closely related hosts, despite operating the same genetic device. We conclude that the functional parameters of the inverter are highly dependent on the host context it is operating within, showing that the basis for the chassis effect stems from unique host-specific properties. We therefore proceeded to characterize the hosts in terms of relevant physiology metrics to encapsulate the unique host conditions each inverter circuit is operating under.

### Physiological diversity among hosts

**Fig. 3.**
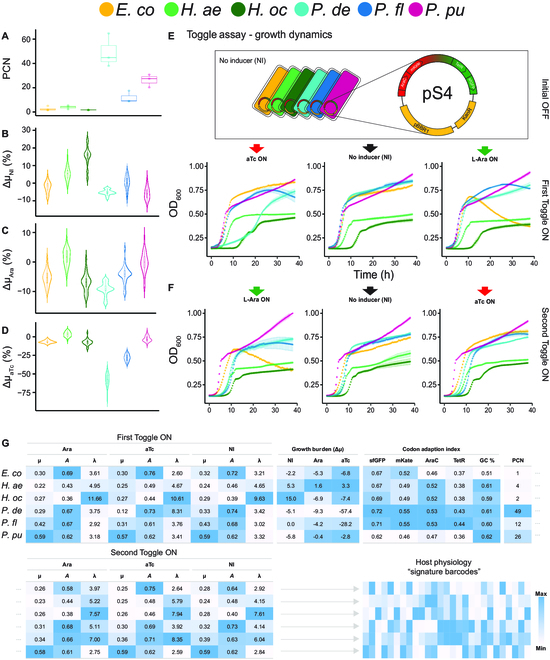
Hosts display a wide range between values among the metrics chosen to capsulate physiology. (A) Estimated PCN in units of plasmid(s) per chromosome (*n* = 3 biological replicates each with technical replicates). Hosts are color-coded; legend at the top of figure. Violin plots of (B) growth burden (Δμ_NI_) in the absence of inducer. A negative Δμ_NI_ value indicates that the pS4 harboring strain has a lower growth rate compared to its WT counterpart. (C) Growth burden of Ara induction (Δμ_Ara_) and (D) aTc induction (Δμ_aTc_) (*n* = 3 biological replicates each with 4 technical replicates). (E) Growth dynamics of hosts from toggling assay experiment toggled from initial OFF to ON state (First Toggle ON) and then (F) to opposite ON state (Second Toggle ON). (G) Summary of quantified physiology metrics for each host. Gray dots denote continuation of table. μ = maximum specific growth rate (h^−1^), *A* = specific carrying capacity (OD_600_) GC % = total genomic GC content. Color scale denoting “Max” and “Min” values is relative for each column.

Host-specific parameters known to affect gene expression were quantified. This include PCN, maximum specific growth rate, carrying capacity, growth burden as a result of propagating and expressing the pS4 plasmid, and the CAI [25,37] for the four coding sequences within the inverter. We uncovered a wide range of physiology metrics among our hosts, suggesting that physiology could explain, and therein possibly be used to predict, differences in performance between bacterial hosts (Fig. 3).

Despite harboring the same origin of replication, a wide range of PCN values was observed among our hosts (Fig. 3A). This shows that PCN is also subject to the chassis effect, as in agreement with previous studies [47,48]. Maintaining a higher number of plasmids has been shown to cause growth inhibition [49], which can be exacerbated upon circuit induction [22], a scenario applicable to the operation of our inverter circuit. We therefore quantified the growth burden of maintaining the inverter in the absence of inducer, given by the Δμ_NI_ metric, which we define as the percent difference in specific growth rate of pS4-carrying strains compared to WT. We also define Δμ_Ara_ and Δμ_aTc_ as the percent difference in growth rate of Ara and aTc induced pS4-carrying strains compared to the growth rate under NI conditions, respectively. The *Pseudomonas* spp. maintained the highest number of plasmid copies, with *P. deceptionensis* peaking at a PCN value of 49 ± 12. In the absence of inducer, *P. deceptionensis* showed slight but consistent reduced growth compared to their WT counterparts (Δμ_NI_ = −5.4 ± 0.9%) (Fig. 3B). Meanwhile, a surprising result was the consistent increase in growth rate by both *Halopseudomonas* spp., with *H. oceani* pS4 having a specific growth rate 15.0% ± 3.4 higher than its WT counterpart despite maintaining an additional plasmid, albeit at a low copy number (PCN = 2 ± 1). Upon induction, *P. deceptionensis* experienced the strongest exacerbation of growth burden, signified by the most negative Δμ_Ara_ and Δμ_aTc_ values of −9.2% ± 0.3 and −58.0% ± 5.0, respectively (Fig 3C and D). *P. fluorescens* was also sensitive to aTc induction, with a Δμ_aTc_ value of −28.2% ± 3.5. The high growth burden of *P. deceptionensis* and *P. fluorescens* is attributable to their high PCN, but *P. putida*, which had the second highest PCN of 26 ± 5, deviated from this trend with Δμ values close to 0. None of the WT strains experienced appreciable growth inhibition when grown in the presence of either inducer (Fig. 1C and D), meaning the source of the observed growth inhibition likely stems from translational load due to resource limitation [50]. The results show that the degree of metabolic burden is unique to each host and can therefore contribute to the chassis effect.

Cell densities (OD_600_) were measured simultaneously during the toggle assay to characterize the specific growth rate, lag time, and carrying capacity of each host (Fig. 3A and B). Both pS4-carrying *Halopseudomonas* spp*.* revealed the lowest specific growth rates and carrying capacities across induction states, consistent with their WT counterparts (Fig. 3G). The growth metrics of the three *Pseudomonas* spp. (Fig. 3G) differed appreciably depending on their past induction history, reinforcing the observed host-specific hysteresis effects on growth phenotypes. For instance, *P. deceptionensis* cells toggled from initial OFF to aTc-ON experienced a long lag phase and reduced specific growth rate. But when cells were toggled from Ara-ON to aTc-ON, the prolonged lag phase and growth rate reduction was alleviated, and growth more closely resembled the dynamics of noninduced control cells. A similar hysteresis effect was observed for *P. fluorescens*. It is notable that despite the low PCN, the two *Halopseudomonas* spp. reached the highest Fss_aTc-mKate_ values in both toggles of mKate ON, 7-fold higher than the Fss_aTc-mKate_ reached by *P. deceptionensis*, the host with the highest PCN. A high PCN therefore does not necessarily result in high Fss fluorescent protein levels. The high Fss_aTc-mKate_ values obtained by the *Halopseudomonas* spp. could be due to lower rate of cell division and associated dilution of the mKate protein, as the two hosts also exhibit the lowest specific growth rates. This theory is supported by the fact that when *P. deceptionensis* was alleviated of the growth inhibition (increased growth rate), the attained Fss_aTc-mKate_ value also decreased 0.4-fold.

We further considered codon usage bias as an innate host property capable of affecting inverter performance, which we parameterized by determining CAI values (range from 0 to 1) for the *sfGFP*, *mKate*, *AraC*, and *TetR* coding sequences. Hosts with a codon usage bias more adapted to the synonymous codon usage in the four coding genes (CAI closer to 1) in the inverter could lead to more efficient translation of gene products, thereby affecting performance and vice versa. *P. deceptionensis* and *P. fluorescens* have the highest CAI values across all four genes (Fig. 3G). *P. putida*, on the other hand, has the lowest CAI values across all four genes, a property it shares with *E. coli* for the *AraC* and *TetR* genes. Previous findings suggest that similar GC content predicts similar codon usage in prokaryotes [51], yet *E. coli*, with the lowest GC content of 51%, has relatively similar CAI values to *Halopseudomonas* and *Pseudomonas* (GC content, 59% to 62%), for all four translated proteins. Our results do not show the expected trend between CAI value and fluorescence output. Still, sequence optimization strategies have been reported to successfully elevate heterologous protein expression [24,52]. We therefore decided to include the calculated CAI values in downstream analysis.

The summarized physiology metrics were abstracted to signature barcodes representing host physiology profiles. The expression of the inverter is inherently coupled to the unique network of metabolic fluxes of each host due to the dependency on host machinery and resources. Given this complexity, it is unlikely that a single variable can reliably predict the performance metrics of our inverter circuit. Considering multiple explanatory variables could provide a more comprehensive prediction of overall performance.

### Significant concordance between host physiology and the chassis effect

The overarching goal of this study was to determine if the chassis effect is more related to differences in host physiology or phylogenomic relatedness. To formally assess the relationship between species-specific differences in performance, physiology, and phylogeny, we employed two multivariate statistical approaches, namely, the Mantel test [53,54] and Procrustes Superimposition (PS) [55–57] analysis. Both tests revealed significant concordance only between host physiology and inverter performance, indicating that the assorted host physiology metrics serve as a more robust predictor of inverter circuit performance than phylogenomic distance-based phylogeny, within our selection of Gammaproteobacteria hosts.

The Mantel test and PS analyses are measures for “goodness-of-fit,” where a high goodness-of-fit indicates that the two datasets are correlated. PS analysis acts on ordinated data by centering, scaling, and superimposing two projected ordination configurations to minimize the vector residuals between each item. The Mantel test considers linear correlation between two distance matrices and produces an R statistic similar to the Pearson's correlation coefficient; with increasingly similar dissimilarity matrices, the Mantel *R* statistic will approach 1. PS analysis produces the *M*^2^ statistic, which is the sum of squared residuals between the best fit of two configurations such that an *M*^2^ value closer to 0 indicates a better fit.

**Fig. 4.**
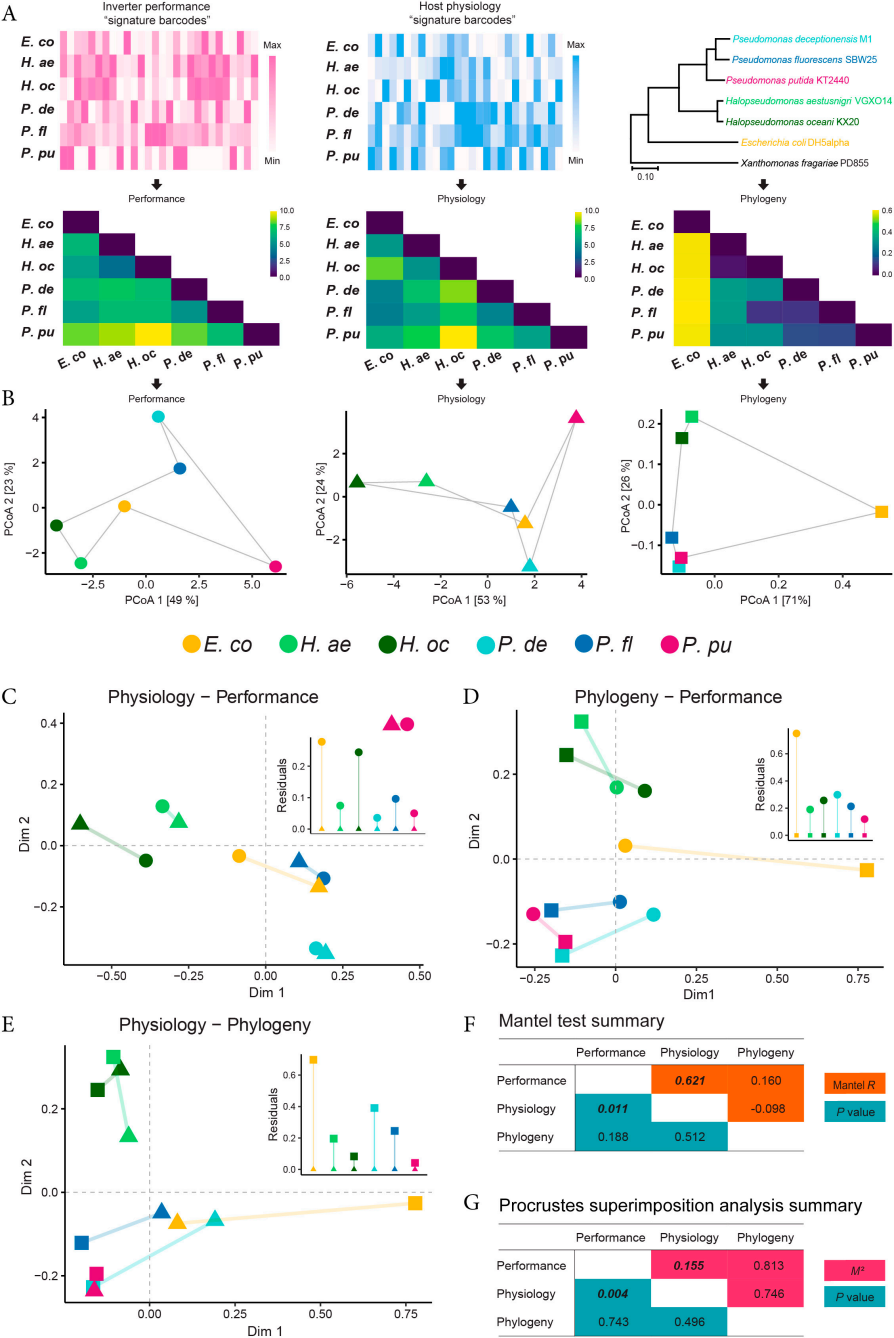
Mantel and PS analysis reveal significant concordance between clustering pattern of hosts in terms of physiology and performance variation. (A) Table of summarized performance (left) and physiology (middle) metrics converted to Euclidean distance metrics (black arrows) with equal weight for each column. Distances in phylogenomic tree from Fig. 1H were converted to a phylogenomic distance matrix that is applicable for Mantel test. (B) PCoA biplots of performance (circles), physiology (triangles), and phylogeny (squares) derived from respective distance matrix. Hosts are color-coded (legend in middle). Solid gray lines help visualize a “configuration” that represents the clustering pattern of hosts for each data type; the lines connect each species in the same arbitrary order. Values in square brackets represent proportion of variation captured by each axis. Symmetric pairwise PS analysis involves scaling, centering, and rotating configurations against each other to minimize vector residuals, fitting (C) phylogeny against performance, (D) physiology against performance, and (E) physiology against phylogeny. Hosts are color-coded, and symbol key follows (B). Colored lines connecting points represent the vector residuals between respective hosts; insets show bar chart of vector residuals. Intersection of dashed gray lines indicates centroid of configurations. Summarized statistics from pairwise (F) Mantel test and (G) PS analysis. Numbers in bold and italics are significant with *P* < 0.05.

To apply the Mantel test, Euclidean distance matrices were generated from the respective physiology and performance metric tables (Fig. 4A), while the phylogenomic tree was converted to a distance matrix format suitable for downstream analysis. The Mantel test revealed a significant positive correlation between performance and physiology distance matrices (Fig. 4F; Mantel *R* = 0.621, *P* = 0.011), indicating that hosts similar in terms of performance are also similar in their overall physiology, and vice versa. When testing performance against phylogeny, no significant correlation was observed (Mantel *R* = 0.160, *P* = 0.188). The Mantel test summarizes the results in a single metric that only describes the general trend, while PS analysis has the advantage in that the contribution of residuals from each individual item (host) is reported. To investigate the result of the Mantel test further, PS analysis was performed to compare the similarity of the ordination configurations of the hosts.

PCoA was applied to distance matrices to project the distances in ordination space (Fig. 4B). As expected, ordination of the phylogenomic distance matrix produces a clustering pattern that reflects the branching pattern of the phylogenomic tree (Fig. 4B, right). *Halopseudomonas* spp*.* clustered across all three types of data; while this is expected in terms of phylogeny, it indicates that they are also similar in terms of their physiology and circuit performance. Pairwise PS analysis confirmed and strengthened our main result, again reporting significant concordance between the clustering pattern of the hosts in terms of observable variance between host-specific physiology and inverter performance (Fig. 4C; *M*^2^ = 0.155, *P* = 0.004), while again no significance was found between phylogeny and performance (Fig. 4D; *M*^2^ = 0.813, *P* = 0.743). Furthermore, neither test found a significant correlation between physiology and phylogeny (Fig. 4E and F), questioning the commonly assumed predictive power of phylogeny for assessing microbial phenotypes or ecophysiology. Closer inspection of each PS comparison revealed an uneven contribution of residuals by each individual host. In the physiology-performance comparison, *E. coli* alone accounts for 36% of the total residual in the data, more than double the theoretical even contribution (16.67%). The second highest contributor of residuals is *H. aestusnigri* with 21% and all other hosts contributing less. This suggests that these 2 hosts do not follow the significant trend (i.e., physiology underpins performance) as strongly as the other hosts. The reason for this unevenness could be because the underlying mechanisms that drive the chassis effect in *E. coli* and *H. aestusnigri* are not included in the analysis. Despite this, the overall low residual was still deemed significant. When comparing phylogeny-performance, *E. coli* is again responsible for the highest proportion of the residual (41%). This suggests that differences in inverter performance are trackable along the phylogenomic tree of species part of the *Pseudomonadaceae* family but lose reliability as they approach *E. coli*.

## Discussion

In this study, we demonstrate within our Gammaproteobacteria framework that the comparative chassis effect on a genetic inverter circuit operated between hosts is better explained by the physiological differences of the host rather than genomic relatedness, confirming our initial hypothesis. To put our findings in context, the performance profile of any new bacterial host transformed with the pS4 plasmid can be inferred by characterizing and comparing its physiology profile against our set of reference hosts. Our results suggest that the new host is likely to have a performance profile similar to a reference host it shares similar physiology profile with, and vice versa. Expanding the reference set of hosts would allow a better statistical inference of device performance based on physiology profiles. We note that the physiology metrics chosen to contextualize our hosts are by no means exhaustive, and that additional insight could be garnered by measurements that reflect the availability of transcriptional and translational machinery and resources. Examples of such metrics are steady-state ribosomal abundance [58], RNA polymerase abundance, and NADH [reduced form of nicotinamide adenine dinucleotide (oxidized form)] pool [59]. However, the measurement of these metrics can be challenging to normalize across hosts [60].

Mechanistic modeling has the advantage of being able to quantitatively predict output parameters, but establishing such a model is challenging or even intractable given that the complexity microbial physiology is compared across species. Our holistic approach has the advantage of being able to consider diverse range of metrics without needing to know how they interact (e.g., growth rate and PCN), under the condition that the inclusion of the metric should be justified with *a priori *insight. Our results push the notion that the biological determinants manifesting a comparable and quantifiable chassis effect can be traced to physiological differences between hosts, and that this holds true for other similar frameworks involving plasmid-based bacterial expression systems. Given the universality of our physiology metrics, our findings could also hold true for eukaryotic expression systems. A major caveat of this result is that a given collection of hosts must also share some base level of genetic compatibility with a given engineered device, therein maintaining some unspecifiable level of dependence upon genetic relatedness. Yet, our results show that the observable chassis effect cannot be reliably inferred from phylogenomic relationships—i.e., genome relatedness. This study also establishes a framework for testing how an engineered genetic circuit might perform across closely related hosts and bolsters momentum in the field of BHR biodesign. Furthermore, to our knowledge, this study is the first to report the successful genetic engineering of three novel marine hosts, *H. aestusnigri* VGOX14, *H. oceani* KX20, and *P. deceptionensis* M1, with pragmatic innate phenotypes such as polyethylene terephthalate (PET) degradation [61], psychrotolerance, and salinity tolerance [62,63].

The diverse range of observed constants across the different chassis was interesting; since both the protein sequences are identical across the data set, changes in kinetic parameters such as the activation constants cannot arise due to differences in binding affinity of the small activating ligands for the transcription factors. Similarly, variations in the Hill coefficient cannot be due to local DNA binding interactions since the promoter sequences are identical. Instead, these chassis-specific effects are more likely due to the expression level of the transcription factor and the bioavailability of the activating ligand, which may also be dependent on transport regulators and efflux. For instance, the markedly higher *K*_aTc_ values for the *Pseudomonas* spp. could be due to the presence of a native TtgABC efflux pump, known to be more highly expressed in the presence of tetracycline in *P. putida* DOT-T1 strain [64,65]. Indeed, a BLAST search with the *P. putida* DOT-T1 *TtgA*, *TtgB*, and *TtgC* genes [National Center for Biotechnology Information (NCBI) GenBank accession = AF031417.2] against the genomes of our six hosts reveals that all three genes are also present in the *P. putida* and *P. fluorescens* genome, the hosts with the highest *K*_aTc_. Active export of the inducer leading to lowered intracellular concentration could explain the wider induction range of *P. putida* and *P. fluorescens*. This is a clear example of how the presence of a single element can be a major contributor to variation. Furthermore, unpredictable elements such as the presence of DNA binding sites on the host genome recognized by the TetR and AraC repressors could lead to sequestration of the transcription factors, leading to differing steady-state concentration of free repressor available to bind to its intended cognate operator [66].

Hysteresis experiments revealed that inverter-carrying hosts initially induced with Ara were unable to stably maintain past fluorescence states in late phase. The reason for the different capacities of hysteresis between the *P*_BAD_ and *P*_Tet_ promoters could be due to the different modes of regulation. The *P*_BAD_ promoter requires Ara-bound AraC to activate transcription; hence, the retention of past induction state is susceptible to negative growth feedback diluting the activator during exponential growth and thereby leading to memory loss. On the other hand, the constitutively active *P*_Tet_ promoter enjoys increased transcription upon dilution of its cognate TetR repressor. This dependence on circuit topology is in agreement with the findings of Zhang et al. [20]. The topology of our inverter circuit differs from the mutual inhibitory motif of classical toggle switches [67,68] not only due to the dual property of the chosen AraC transcription factor, but also because the *P*_BAD_ promoter contains a catabolite activator protein binding site, leading to a potential topology change as cells exit exponential growth and enter slow growth conditions. Catabolite repression effect could therefore also contribute to the inability of host cells to retain a stable sfGFP ON phenotype. As the purpose of the study was to characterize the chassis effect and not the inverter circuit, we did not conduct an extensive investigation of the inverter’s complex dynamics.

The chassis effect can manifest in different ways between hosts, from complete circuit inoperability in some hosts, to variations in performance between others. The diversity of performance metric profiles exhibited by the six hosts characterized here encourages the notion of viewing chassis as pragmatic parts capable of tuning device parameters. In cases where the tuning of promoter or ribosome binding site (RBS) strengths does not yield the desired functional outcome, exploring chassis design space could be a promising direction. However, we recognize that successfully introducing a device into a novel chassis is often not possible; as exemplified during our design–build–test processes, the pS4 plasmid was unsuccessfully transformed into two initially chosen hosts: *Pseudomonas taeanensis* MS-3 and *Halopseudomonas pachastrellae* CCUG 46540 (NCBI Assembly accession numbers MS-3 and ASM198937v1, respectively). Whether this was due to transformation methodology, genetic compatibility, or the activity of inverter circuit being toxic to the cells is unknown, indicating that the “inoperable chassis effect” is challenging to assess.

Armed with automated circuit design software [69], standardized part libraries, and robust DNA assembly technology, synthetic biologists are now able to reliably manifest their genetic circuit abstractions into physical DNA molecules that carry out programmed functions with increasing fidelity. We have improved our ability to control for unwanted compositional and context effects [17] in our circuit designs to a high degree by compartmentalizing modules and components using regulatory elements such as terminators and ribozymes [70] and rationally avoiding promiscuous genetic elements known to “cross-talk.” However, continued reliance on our limited number of model organisms will surely stagnate the rate of progress of engineering microorganisms. As we domesticate more pragmatic microbes as novel chassis, our ability to control and predict host context effect must also advance. The emerging field of BHR synthetic biology aims to develop engineering principles that minimize unpredictability arising from host context, and our findings contribute to this goal by improving prediction of the chassis effect; we have here uncovered potential fundamental principles that drive observable differences in host-specific genetic device performance, and thereby lending increased predictive power for porting synthetic circuits across bacterial species and the exploration of chassis design space.

## Data Availability

All experimental data files, raw data files, and R MarkDown scripts used for analysis and plotting are publicly available online on the Open Science Framework as part of the project name *Chan.Interspecies.Inverter.Circuit* (https://osf.io/jnkrx/). Genome, plasmid, and bacterial strain accession numbers can be found in the Supplementary Materials.

## Supplementary Materials

Supplementary 1 Figs. S1 and S2 Tables S1 to S5 References [71–81]
