# Supplementary material for: Revealing the Host-Dependent Nature of an Engineered Genetic Inverter in Concordance with Physiology
Source: Biodes Res. 2023 Aug 16;5:0016. doi: 10.34133/bdr.0016 (PMC10432152; doi:10.34133/bdr.0016)
Supplement: Supplementary 1 — Figs. S1 and S2 Tables S1 to S5 References [71–81] [file bdr.0016.f1.zip › SM.docx]

# SUPPLEMENTARY MATERIAL

**
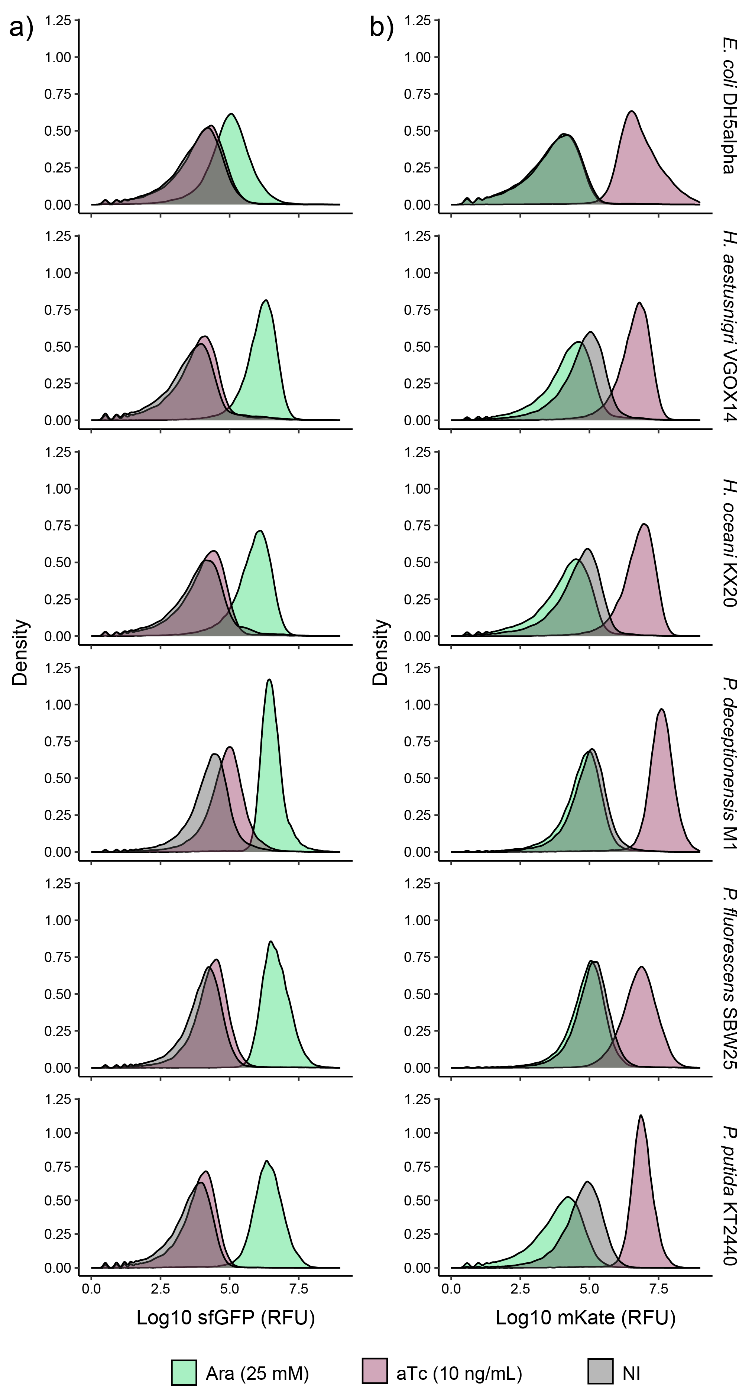
**

**Supplementary Figure S1. Inverter-carrying hosts achieves uniform fluorescence states upon induction.** a) sfGFP and corresponding b) mKate fluorescence distribution of host populations across induction states at late exponential phase. 20 000 events were recorded per sample. NI = No inducer, n = 4.

**
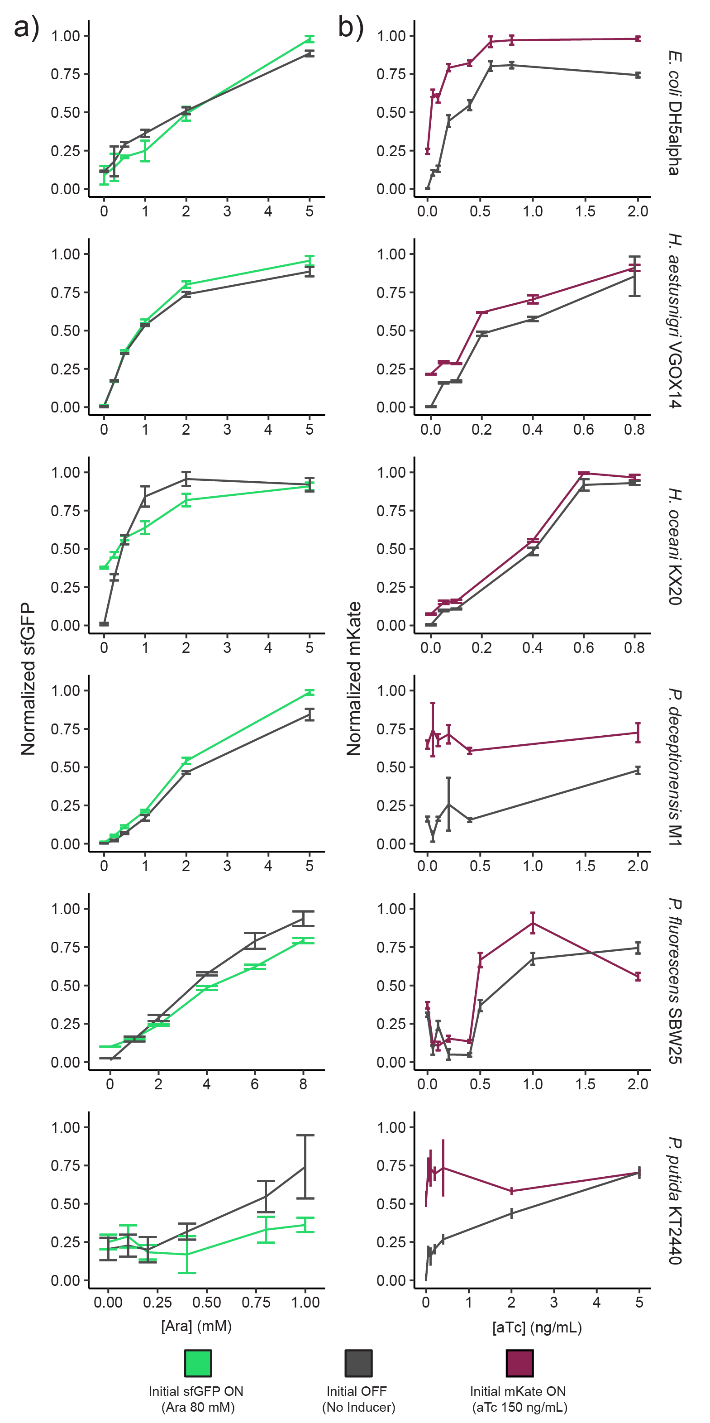
**

**Supplementary Figure S2. Inverter-carrying cells are unable to retain sfGFP ON phenotype.** a) sfGFP and b) mKate hysteresis curves of cells pretreated with 80 mM L-Arabinose (Initial sfGFP ON), 150 ng/mL anhydrotetracycline (Initial mKate ON) and no inducer (Initial OFF). Fluorescence is normalized against the highest fluorescence value for each inducer and host. For certain hosts initially oversaturated with aTc, there is a clear range in which the same concentration of inducer produces different fluorescence output, indicative of hysteresis effect. n = 4.

**Supplementary Table S1.** Single copy gene hits of the 172 Gammaproteobacteria Hidden Markov Models in the GToTree Program and report statistics for each species used in this study. The number of hits for each gene is indicated.

|  | **Host** | Pseudomonas fluorescens | Pseudomonas putida | Pseudomonas deceptionensis | Halopseudomonas aestusnigri | Escherichia  coli | Halopseudomonas oceani | Xanthomonas fragariae |
| --- | --- | --- | --- | --- | --- | --- | --- | --- |
|  | **num_SCG_hits** | 166 | 171 | 172 | 172 | 172 | 171 | 167 |
|  | **uniq_SCG_hits** | 159 | 167 | 171 | 170 | 172 | 167 | 167 |
|  | **perc_comp** | 97 | 99 | 100 | 100 | 100 | 99 | 97 |
|  | **perc_redund** | 4.7 | 2.3 | 0.6 | 1.2 | 0 | 2.9 | 0 |
|  | **num_SCG_hits_after_len_filt** | 159 | 166 | 170 | 170 | 166 | 167 | 159 |
|  | **in_final_tree** | Yes | Yes | Yes | Yes | Yes | Yes | Yes |
| **PFAM Accession Number:** | **NCBI_species** | Pseudomonas fluorescens | Pseudomonas putida | Pseudomonas deceptionensis | Halopseudomonas aestusnigri | Escherichia coli | Halopseudomonas oceani | Xanthomonas fragariae |
| **PF01812.20** | **5-FTHF_cyc-lig** | **1** | **1** | **1** | **1** | **1** | **1** | **1** |
| **PF00022.19** | **Actin** | **1** | **1** | **1** | **1** | **1** | **1** | **1** |
| **PF00709.21** | **Adenylsucc_synt** | **1** | **1** | **1** | **1** | **1** | **1** | **1** |
| **PF00406.22** | **ADK** | **1** | **1** | **1** | **1** | **1** | **1** | **1** |
| **PF01808.18** | **AICARFT_IMPCHas** | **1** | **1** | **1** | **1** | **1** | **1** | **1** |
| **PF00731.20** | **AIRC** | **1** | **1** | **1** | **1** | **1** | **1** | **1** |
| **PF00490.21** | **ALAD** | **2** | **2** | **1** | **1** | **1** | **1** | **1** |
| **PF03702.14** | **AnmK** | **1** | **1** | **1** | **1** | **1** | **1** | **1** |
| **PF00231.19** | **ATP-synt** | **1** | **1** | **1** | **1** | **1** | **1** | **1** |
| **PF00119.20** | **ATP-synt_A** | **1** | **1** | **1** | **1** | **1** | **1** | **1** |
| **PF00430.18** | **ATP-synt_B** | **1** | **1** | **1** | **1** | **1** | **1** | **1** |
| **PF00137.21** | **ATP-synt_C** | **1** | **1** | **1** | **1** | **1** | **1** | **1** |
| **PF02823.16** | **ATP-synt_DE_N** | **1** | **1** | **1** | **1** | **1** | **1** | **1** |
| **PF03899.15** | **ATP-synt_I** | **1** | **1** | **1** | **1** | **1** | **1** | **0** |
| **PF03668.15** | **ATP_bind_2** | **1** | **1** | **1** | **1** | **1** | **1** | **1** |
| **PF04380.13** | **BMFP** | **1** | **1** | **1** | **1** | **1** | **1** | **1** |
| **PF01264.21** | **Chorismate_synt** | **1** | **1** | **1** | **1** | **1** | **1** | **1** |
| **PF02674.16** | **Colicin_V** | **1** | **1** | **1** | **1** | **1** | **1** | **1** |
| **PF01218.18** | **Coprogen_oxidas** | **1** | **1** | **1** | **1** | **1** | **1** | **1** |
| **PF00166.21** | **Cpn10** | **1** | **1** | **1** | **1** | **1** | **1** | **1** |
| **PF00118.24** | **Cpn60_TCP1** | **1** | **1** | **1** | **1** | **1** | **1** | **1** |
| **PF05173.14** | **DapB_C** | **1** | **1** | **1** | **1** | **1** | **1** | **1** |
| **PF01678.19** | **DAP_epimerase** | **3** | **2** | **1** | **1** | **1** | **1** | **1** |
| **PF01761.20** | **DHQ_synthase** | **1** | **1** | **1** | **1** | **1** | **1** | **1** |
| **PF04977.15** | **DivIC** | **1** | **1** | **1** | **1** | **1** | **1** | **1** |
| **PF00885.19** | **DMRL_synthase** | **2** | **1** | **2** | **2** | **1** | **2** | **1** |
| **PF04364.13** | **DNA_pol3_chi** | **1** | **1** | **1** | **1** | **1** | **1** | **1** |
| **PF06144.13** | **DNA_pol3_delta** | **1** | **1** | **1** | **1** | **1** | **1** | **1** |
| **PF02622.15** | **DUF179** | **1** | **1** | **1** | **1** | **1** | **1** | **1** |
| **PF04241.15** | **DUF423** | **1** | **1** | **1** | **1** | **1** | **1** | **1** |
| **PF04356.12** | **DUF489** | **1** | **1** | **1** | **1** | **1** | **1** | **1** |
| **PF04359.14** | **DUF493** | **1** | **1** | **1** | **1** | **1** | **1** | **1** |
| **PF04751.14** | **DUF615** | **1** | **1** | **1** | **1** | **1** | **1** | **1** |
| **PF00889.19** | **EF_TS** | **1** | **1** | **1** | **1** | **1** | **1** | **1** |
| **PF01176.19** | **eIF-1a** | **1** | **1** | **1** | **1** | **1** | **1** | **1** |
| **PF00113.22** | **Enolase_C** | **2** | **1** | **1** | **1** | **1** | **1** | **1** |
| **PF02601.15** | **Exonuc_VII_L** | **1** | **1** | **1** | **1** | **1** | **1** | **1** |
| **PF02609.16** | **Exonuc_VII_S** | **1** | **1** | **1** | **1** | **1** | **1** | **1** |
| **PF00762.19** | **Ferrochelatase** | **1** | **1** | **1** | **1** | **1** | **1** | **1** |
| **PF04999.13** | **FtsL** | **1** | **1** | **1** | **1** | **1** | **1** | **1** |
| **PF04551.14** | **GcpE** | **1** | **1** | **1** | **1** | **1** | **1** | **1** |
| **PF01025.19** | **GrpE** | **1** | **1** | **1** | **1** | **1** | **1** | **1** |
| **PF01725.16** | **Ham1p_like** | **1** | **1** | **1** | **1** | **1** | **1** | **1** |
| **PF02602.15** | **HEM4** | **1** | **1** | **1** | **2** | **1** | **2** | **1** |
| **PF17209.3** | **Hfq** | **1** | **1** | **1** | **1** | **1** | **1** | **1** |
| **PF01634.18** | **HisG** | **1** | **1** | **1** | **1** | **1** | **1** | **1** |
| **PF00815.20** | **Histidinol_dh** | **2** | **1** | **1** | **1** | **1** | **1** | **1** |
| **PF01430.19** | **HSP33** | **1** | **1** | **1** | **1** | **1** | **1** | **1** |
| **PF00475.18** | **IGPD** | **1** | **1** | **1** | **1** | **1** | **1** | **1** |
| **PF01715.17** | **IPPT** | **1** | **1** | **1** | **1** | **1** | **1** | **1** |
| **PF01745.16** | **IPT** | **1** | **1** | **1** | **1** | **1** | **0** | **1** |
| **PF04362.14** | **Iron_traffic** | **1** | **1** | **1** | **1** | **1** | **1** | **1** |
| **PF06305.11** | **LapA_dom** | **0** | **1** | **1** | **1** | **1** | **1** | **1** |
| **PF03588.14** | **Leu_Phe_trans** | **1** | **1** | **1** | **1** | **1** | **1** | **1** |
| **PF03550.14** | **LolB** | **1** | **1** | **1** | **1** | **1** | **1** | **1** |
| **PF02684.15** | **LpxB** | **1** | **1** | **1** | **1** | **1** | **1** | **1** |
| **PF03331.13** | **LpxC** | **1** | **1** | **1** | **1** | **1** | **1** | **1** |
| **PF02606.14** | **LpxK** | **1** | **1** | **1** | **1** | **1** | **1** | **1** |
| **PF02401.18** | **LYTB** | **1** | **1** | **1** | **1** | **1** | **1** | **1** |
| **PF03776.14** | **MinE** | **1** | **1** | **1** | **1** | **1** | **1** | **1** |
| **PF05494.12** | **MlaC** | **1** | **1** | **1** | **1** | **1** | **1** | **1** |
| **PF12631.7** | **MnmE_helical** | **1** | **1** | **1** | **1** | **1** | **1** | **1** |
| **PF04085.14** | **MreC** | **1** | **1** | **1** | **1** | **1** | **1** | **1** |
| **PF04093.12** | **MreD** | **1** | **1** | **1** | **1** | **1** | **1** | **1** |
| **PF00334.19** | **NDK** | **1** | **1** | **1** | **1** | **1** | **1** | **1** |
| **PF03938.14** | **OmpH** | **1** | **1** | **1** | **1** | **1** | **1** | **0** |
| **PF00213.18** | **OSCP** | **1** | **1** | **1** | **1** | **1** | **1** | **1** |
| **PF02569.15** | **Pantoate_ligase** | **1** | **1** | **1** | **1** | **1** | **1** | **1** |
| **PF02153.17** | **PDH** | **2** | **1** | **1** | **1** | **1** | **1** | **1** |
| **PF00800.18** | **PDT** | **1** | **1** | **1** | **1** | **1** | **1** | **1** |
| **PF03740.13** | **PdxJ** | **1** | **1** | **1** | **1** | **1** | **1** | **1** |
| **PF00311.17** | **PEPcase** | **1** | **1** | **1** | **1** | **1** | **1** | **1** |
| **PF01252.18** | **Peptidase_A8** | **1** | **1** | **1** | **1** | **1** | **3** | **1** |
| **PF01195.19** | **Pept_tRNA_hydro** | **1** | **1** | **1** | **1** | **1** | **1** | **1** |
| **PF00342.19** | **PGI** | **1** | **2** | **1** | **1** | **1** | **1** | **1** |
| **PF00162.19** | **PGK** | **1** | **1** | **1** | **1** | **1** | **1** | **1** |
| **PF03831.14** | **PhnA** | **1** | **1** | **1** | **1** | **1** | **1** | **1** |
| **PF02233.16** | **PNTB** | **1** | **1** | **1** | **1** | **1** | **1** | **0** |
| **PF01379.20** | **Porphobil_deam** | **1** | **1** | **1** | **1** | **1** | **1** | **1** |
| **PF00697.22** | **PRAI** | **1** | **1** | **1** | **1** | **1** | **1** | **1** |
| **PF01255.19** | **Prenyltransf** | **1** | **1** | **1** | **1** | **1** | **1** | **1** |
| **PF01416.20** | **PseudoU_synth_1** | **1** | **1** | **1** | **1** | **1** | **1** | **1** |
| **PF02666.15** | **PS_Dcarbxylase** | **1** | **1** | **1** | **1** | **1** | **1** | **1** |
| **PF02033.18** | **RBFA** | **1** | **1** | **1** | **1** | **1** | **1** | **1** |
| **PF02565.15** | **RecO_C** | **1** | **1** | **1** | **1** | **1** | **1** | **1** |
| **PF02631.16** | **RecX** | **1** | **1** | **1** | **1** | **1** | **1** | **1** |
| **PF00825.18** | **Ribonuclease_P** | **0** | **1** | **1** | **1** | **1** | **1** | **1** |
| **PF00687.21** | **Ribosomal_L1** | **1** | **1** | **1** | **1** | **1** | **1** | **1** |
| **PF00572.18** | **Ribosomal_L13** | **1** | **1** | **1** | **1** | **1** | **1** | **1** |
| **PF00238.19** | **Ribosomal_L14** | **1** | **1** | **1** | **1** | **1** | **1** | **1** |
| **PF00252.18** | **Ribosomal_L16** | **1** | **1** | **1** | **1** | **1** | **1** | **1** |
| **PF01196.19** | **Ribosomal_L17** | **1** | **1** | **1** | **1** | **1** | **1** | **1** |
| **PF00861.22** | **Ribosomal_L18p** | **1** | **1** | **1** | **1** | **1** | **1** | **1** |
| **PF01245.20** | **Ribosomal_L19** | **1** | **1** | **1** | **1** | **1** | **1** | **1** |
| **PF00453.18** | **Ribosomal_L20** | **1** | **1** | **1** | **1** | **1** | **1** | **1** |
| **PF00829.21** | **Ribosomal_L21p** | **1** | **1** | **1** | **1** | **1** | **1** | **1** |
| **PF00237.19** | **Ribosomal_L22** | **1** | **1** | **1** | **1** | **1** | **1** | **1** |
| **PF00276.20** | **Ribosomal_L23** | **1** | **1** | **1** | **1** | **1** | **1** | **1** |
| **PF17136.4** | **ribosomal_L24** | **1** | **1** | **1** | **1** | **1** | **1** | **1** |
| **PF01016.19** | **Ribosomal_L27** | **1** | **1** | **1** | **1** | **1** | **1** | **1** |
| **PF00828.19** | **Ribosomal_L27A** | **1** | **1** | **1** | **1** | **1** | **1** | **1** |
| **PF00830.19** | **Ribosomal_L28** | **1** | **1** | **1** | **1** | **1** | **1** | **1** |
| **PF00831.23** | **Ribosomal_L29** | **1** | **1** | **1** | **1** | **1** | **1** | **1** |
| **PF00297.22** | **Ribosomal_L3** | **1** | **1** | **1** | **1** | **1** | **1** | **1** |
| **PF01783.23** | **Ribosomal_L32p** | **1** | **1** | **1** | **1** | **1** | **1** | **1** |
| **PF00471.20** | **Ribosomal_L33** | **1** | **1** | **1** | **1** | **1** | **1** | **1** |
| **PF00468.17** | **Ribosomal_L34** | **0** | **1** | **1** | **1** | **1** | **1** | **1** |
| **PF01632.19** | **Ribosomal_L35p** | **1** | **1** | **1** | **1** | **1** | **1** | **1** |
| **PF00573.22** | **Ribosomal_L4** | **1** | **1** | **1** | **1** | **1** | **1** | **1** |
| **PF00347.23** | **Ribosomal_L6** | **1** | **1** | **1** | **1** | **1** | **1** | **1** |
| **PF03948.14** | **Ribosomal_L9_C** | **1** | **1** | **1** | **1** | **1** | **1** | **1** |
| **PF00338.22** | **Ribosomal_S10** | **1** | **1** | **1** | **1** | **1** | **1** | **1** |
| **PF00411.19** | **Ribosomal_S11** | **1** | **1** | **1** | **1** | **1** | **1** | **1** |
| **PF00416.22** | **Ribosomal_S13** | **1** | **1** | **1** | **1** | **1** | **1** | **1** |
| **PF00253.21** | **Ribosomal_S14** | **1** | **1** | **1** | **1** | **1** | **1** | **1** |
| **PF00312.22** | **Ribosomal_S15** | **1** | **1** | **1** | **1** | **1** | **1** | **1** |
| **PF00886.19** | **Ribosomal_S16** | **1** | **1** | **1** | **1** | **1** | **1** | **1** |
| **PF00366.20** | **Ribosomal_S17** | **1** | **1** | **1** | **1** | **1** | **1** | **1** |
| **PF00203.21** | **Ribosomal_S19** | **1** | **1** | **1** | **1** | **1** | **1** | **1** |
| **PF00318.20** | **Ribosomal_S2** | **1** | **1** | **1** | **1** | **1** | **1** | **1** |
| **PF01649.18** | **Ribosomal_S20p** | **1** | **1** | **1** | **1** | **1** | **1** | **1** |
| **PF01165.20** | **Ribosomal_S21** | **1** | **1** | **1** | **1** | **1** | **1** | **1** |
| **PF01250.17** | **Ribosomal_S6** | **1** | **1** | **1** | **1** | **1** | **1** | **1** |
| **PF00177.21** | **Ribosomal_S7** | **1** | **1** | **1** | **1** | **1** | **1** | **1** |
| **PF00410.19** | **Ribosomal_S8** | **1** | **1** | **1** | **1** | **1** | **1** | **1** |
| **PF00380.19** | **Ribosomal_S9** | **1** | **1** | **1** | **1** | **1** | **1** | **1** |
| **PF00164.25** | **Ribosom_S12_S23** | **1** | **1** | **1** | **1** | **1** | **1** | **1** |
| **PF06026.14** | **Rib_5-P_isom_A** | **1** | **1** | **1** | **1** | **1** | **1** | **1** |
| **PF02646.16** | **RmuC** | **1** | **1** | **1** | **1** | **1** | **1** | **1** |
| **PF01351.18** | **RNase_HII** | **1** | **1** | **1** | **1** | **1** | **1** | **1** |
| **PF01193.24** | **RNA_pol_L** | **1** | **1** | **1** | **1** | **1** | **1** | **1** |
| **PF01192.22** | **RNA_pol_Rpb6** | **1** | **1** | **1** | **1** | **1** | **1** | **1** |
| **PF01765.19** | **RRF** | **1** | **1** | **1** | **1** | **1** | **1** | **1** |
| **PF02410.15** | **RsfS** | **1** | **1** | **1** | **1** | **1** | **1** | **1** |
| **PF02075.17** | **RuvC** | **1** | **1** | **1** | **1** | **1** | **1** | **1** |
| **PF03652.15** | **RuvX** | **1** | **1** | **1** | **1** | **1** | **1** | **1** |
| **PF01259.18** | **SAICAR_synt** | **1** | **1** | **1** | **1** | **1** | **1** | **1** |
| **PF04445.13** | **SAM_MT** | **1** | **1** | **1** | **1** | **1** | **1** | **0** |
| **PF03937.16** | **Sdh5** | **1** | **1** | **1** | **1** | **1** | **1** | **1** |
| **PF02556.14** | **SecB** | **1** | **1** | **1** | **1** | **1** | **1** | **1** |
| **PF00584.20** | **SecE** | **0** | **1** | **1** | **1** | **1** | **1** | **1** |
| **PF03840.14** | **SecG** | **0** | **1** | **1** | **1** | **1** | **1** | **1** |
| **PF00344.20** | **SecY** | **1** | **1** | **1** | **1** | **1** | **1** | **1** |
| **PF04102.12** | **SlyX** | **1** | **1** | **1** | **1** | **1** | **1** | **1** |
| **PF01668.18** | **SmpB** | **1** | **1** | **1** | **1** | **1** | **1** | **1** |
| **PF02590.17** | **SPOUT_MTase** | **1** | **1** | **1** | **1** | **1** | **1** | **1** |
| **PF04386.13** | **SspB** | **1** | **1** | **1** | **1** | **1** | **1** | **1** |
| **PF00902.18** | **TatC** | **1** | **2** | **1** | **1** | **1** | **1** | **1** |
| **PF01702.18** | **TGT** | **1** | **1** | **1** | **1** | **1** | **1** | **1** |
| **PF00303.19** | **Thymidylat_synt** | **1** | **1** | **1** | **1** | **1** | **1** | **1** |
| **PF03966.16** | **Trm112p** | **1** | **1** | **1** | **1** | **1** | **1** | **1** |
| **PF00750.19** | **tRNA-synt_1d** | **1** | **1** | **1** | **1** | **1** | **1** | **1** |
| **PF01411.19** | **tRNA-synt_2c** | **2** | **1** | **1** | **1** | **1** | **1** | **1** |
| **PF02091.15** | **tRNA-synt_2e** | **1** | **1** | **1** | **1** | **1** | **1** | **1** |
| **PF01746.21** | **tRNA_m1G_MT** | **1** | **1** | **1** | **1** | **1** | **1** | **1** |
| **PF02092.17** | **tRNA_synt_2f** | **1** | **1** | **1** | **1** | **1** | **1** | **1** |
| **PF02580.16** | **Tyr_Deacylase** | **1** | **1** | **1** | **1** | **1** | **1** | **1** |
| **PF03658.14** | **Ub-RnfH** | **1** | **0** | **1** | **1** | **1** | **1** | **1** |
| **PF02130.17** | **UPF0054** | **1** | **1** | **1** | **1** | **1** | **1** | **1** |
| **PF02021.17** | **UPF0102** | **1** | **1** | **1** | **1** | **1** | **1** | **1** |
| **PF17775.1** | **UPF0225** | **1** | **1** | **1** | **1** | **1** | **1** | **1** |
| **PF14681.6** | **UPRTase** | **1** | **1** | **1** | **1** | **1** | **2** | **1** |
| **PF01208.17** | **URO-D** | **1** | **1** | **1** | **1** | **1** | **1** | **1** |
| **PF02699.15** | **YajC** | **1** | **1** | **1** | **1** | **1** | **1** | **1** |
| **PF02575.16** | **YbaB_DNA_bd** | **1** | **1** | **1** | **1** | **1** | **1** | **1** |
| **PF02620.17** | **YceD** | **1** | **1** | **1** | **1** | **1** | **1** | **1** |
| **PF02618.16** | **YceG** | **1** | **1** | **1** | **1** | **1** | **1** | **1** |
| **PF05166.13** | **YcgL** | **1** | **1** | **1** | **1** | **1** | **1** | **1** |
| **PF02542.16** | **YgbB** | **1** | **1** | **1** | **1** | **1** | **1** | **1** |
| **PF02325.17** | **YGGT** | **1** | **1** | **1** | **1** | **1** | **1** | **0** |
| **PF03755.13** | **YicC_N** | **1** | **1** | **1** | **1** | **1** | **1** | **1** |
| **PF01809.18** | **YidD** | **0** | **1** | **1** | **1** | **1** | **1** | **1** |

**Supplementary Table S2.** Metadata on strains used in this study. DSM strain number and NCBI Assembly accession numbers provided are the latest at the time of the publication of this study.

| **Species** | **Genotype** | **Culture Collection*** | **NCBI Accession** | **Ref.** |
| --- | --- | --- | --- | --- |
| *Halopseudomonas aestusnigri* VGXO14 | WT | DSMZ (103065) | ASM219798v1 | ^71,72^ |
| *Pseudomonas deceptionensis* M1 | WT | DSMZ (26521) | G8684 | ^63,73^ |
| *Pseudomonas fluorescens* SBW25 | WT | Donated** | MPBAS00001 | ^74^ |
| *Halopseudomonas oceani* KX20 | WT | DSMZ (100277) | ASM290316v1 | ^62,75^ |
| *Pseudomonas putida* KT2440 | WT | DSMZ (6125) | ASM756v2 | ^76^ |
| *Escherichia coli* DH5α | WT | DSMZ (6897) | ASM289947v1 | ^77^ |
| *Xanthomonas fragariae* PD855 | WT | Na | PD885.1 | ^78^ |
| *Halopseudomonas aestusnigri* VGXO14 | pS4 | This study | This study | This study |
| *Pseudomonas deceptionensis* M1 | pS4 | This study | This study | This study |
| *Pseudomonas fluorescens* SBW25 | pS4 | This study | This study | This study |
| *Halopseudomonas oceani* KX20 | pS4 | This study | This study | This study |
| *Pseudomonas putida* KT2440 | pS4 | This study | This study | This study |
| *Escherichia coli* DH5α | pS4 | This study | This study | This study |

*DSMZ = German Collection of Microorganisms and Cell Cultures. Numbers in brackets are DSMZ accession numbers.

**Donation by Rosemarie Wilton, Argonne National Laboratory

**Supplementary Table S3**. Primers used in this study.

| Name | Function | Sequence (5’-3’) | Ref. |
| --- | --- | --- | --- |
| B_pSEVA_F | Integration of pSEVA vectors to BASIC format | TCTGGTGGGTCTCTGTCCAC  TAGTCTTGGACTCCTGTTG | This study |
| B_pSEVA_R | Integration of pSEVA vectors to BASIC format | CGATAGGTCTCCCGAGCCTTAATTAAAGG  CATCAAATAAAACGAAAGGC | This study |
| V-L1-F | Assembly verification and Sanger sequencing | GACACTCCGAGACAGTCAGAGGGTA | ^29^ |
| V-L1-R | Assembly verification and Sanger sequencing | TACCCTCTGACTGTCTCGGAGTGTC | ^29^ |
| V-L2-F | Assembly verification and Sanger sequencing | GTGTGAAAAGTCAGTATCCAGTCGTGTAGTTC | ^29^ |
| V-L2-R | Assembly verification and Sanger sequencing | GAACTACACGACTGGATACTGACTTTTCACAC | ^29^ |
| V-LMiP-F | Assembly verification and Sanger sequencing | CGTGGAAACACTATTATCTGGTGGG | ^29^ |
| V-LMiP-R | Assembly verification and Sanger sequencing | CCCACCAGATAATAGTGTTTCCACG | ^29^ |
| V-LMiS-F | Assembly verification and Sanger sequencing | CAGTCCAATCTGGTGTAACTTCGG | ^29^ |
| V-LMiS-R | Assembly verification and Sanger sequencing | CCGAAGTTACACCAGATTGGACTG | ^29^ |
| V-UTR1-F | Assembly verification and Sanger sequencing | CACCGTCTCAGGTAAGTATCAG | ^29^ |
| V-UTR1-S | Assembly verification and Sanger sequencing | CTGATACTTACCTGAGACGGTG | ^29^ |
| V-UTR2-F | Assembly verification and Sanger sequencing | CTATTGGCTGAGATAAGGGTAGC | ^29^ |
| V-UTR2-S | Assembly verification and Sanger sequencing | GCTACCCTTATCTCAGCCAATAG | ^29^ |
| V-UTR3-F | Assembly verification and Sanger sequencing | CTCGTGGTCTGACGGTAAAATC | ^29^ |
| V-UTR3-R | Assembly verification and Sanger sequencing | GATTTTACCGTCAGACCACGAG | ^29^ |
| Kan_bla_q_F | Plasmid copy number determination | GAACTGGCTGACCGAATTTATG | This study |
| Kan_bla_q_R | Plasmid copy number determination | TATTCTTCCAGCACCTGAAACG | This study |
| E.co_rpoD_q_F | Plasmid copy number determination | TATCAACCGTCGTATGTCCATC | This study |
| E.co_rpoD_q_R | Plasmid copy number determination | AATCAGGTCAAGGAACTGCAAG | This study |
| P.ae_rpoD_q_F | Plasmid copy number determination | AGGAAGGCATCCGTGAAGTC | This study |
| P.ae_rpoD_q_R | Plasmid copy number determination | GATATAGCCGCTGAAAATGTCG | This study |
| P.de_rpoD_q_F | Plasmid copy number determination | CGTTCTGAGCGGTTATATCGAC | This study |
| P.de_rpoD_q_R | Plasmid copy number determination | CGGTGGACTCTTCCTTATCATC | This study |
| P.oc_rpoD_q_F | Plasmid copy number determination | ATGACGAGGAAGAAGACGATTC | This study |
| P.oc_rpoD_q_R | Plasmid copy number determination | CACCTTCTGCAACTTCTCCAG | This study |
| P.pu_rpoD_q_F | Plasmid copy number determination | GCACTCTCGAATACGTTGATCC | This study |
| P.pu_rpoD_q_R | Plasmid copy number determination | TGAGCAGGGCTACCTGACTTAC | This study |

**Supplementary Table S4.** DNA parts and BASIC linkers used in BASIC assembly of pS4 plasmid this study. Accession numbers provided are the latest at the time of the publication of this study.

| **Gene/Part** | **Function** | **NCBI Accession** | **Cloning** | **Ref.** |
| --- | --- | --- | --- | --- |
| sfGFP | Fluorescent reporter gene | MG323875 | Synthesized from BioTwist | ^79^ |
| mKate | Fluorescent reporter gene | MN623117 | Synthesized from BioTwist | ^79^ |
| AraC | Gene encoding L-Ara inducible repressor | MH101733 | Synthesized from BioTwist | ^80^ |
| TetR | Gene encoding aTc inducible repressor | MH101732 | Synthesized from BioTwist | ^80^ |
| B_23 | BASIC integrated pSEVA231 backbone with pBBR1 *ori* and Kan^R^ marker | JX560328 | Cloned with B_pSEVA_F and B_pSEVA_R primers | ^81^ |
| P27_PF1 | Insulated P_Tet_ promoter with upstream terminator and riboJ autocatalytic ribozyme | NA | See ref | ^30^ |
| P32_PF1 | Insulated P_BAD_ promoter with upstream terminator and riboJ autocatalytic ribozyme | NA | See ref | ^30^ |
| BASIC Linkers | BASIC assembly of parts and adding ribosome binding sites | NA | Purchased from BIOLEGIO (BBT-18500) | ^29^ |

**Supplementary Table S5.** DNA Sequence of major components of inverter circuit.

| Part Name | Sequence |
| --- | --- |
| P_Tet_ | TTTTCAGCAGGACGCACTGACCTCCCTATCAGTGATAGAGATTGACATCC |
| AraC | ATGGCTGAAGCGCAAAATGATCCCCTGCTGCCGGGATACTCGTTTAATGCCCATCTGGTGGCGGGTTTAACGCCGATTGAGGCCAACGGTTATCTCGATTTTTTTATCGACCGACCGCTGGGAATGAAAGGTTATATTCTCAATCTCACCATTCGCGGTCAGGGGGTGGTGAAAAATCAGGGACGAGAATTTGTTTGCCGACCGGGTGATATTTTGCTGTTCCCGCCAGGAGAGATTCATCACTACGGTCGTCATCCGGAGGCTCGCGAATGGTATCACCAGTGGGTTTACTTTCGTCCGCGCGCCTACTGGCATGAATGGCTTAACTGGCCGTCAATATTTGCCAATACGGGGTTCTTTCGCCCGGATGAAGCGCACCAGCCGCATTTCAGCGACTTTTTTGGGCAAATCATTAACGCCGGGCAAGGGGAAGGGCGCTATTCGGAGCTGCTGGCGATAAATCTGCTTGAGCAATTGTTACTGCGGCGCATGCTAGCGATTAACGGATCGCTCCATCCACCGATGGATAATCGGGTACGCGAGGCTTGTCAGTACATCAGCGATCACCTGGCAGACAGCAATTTTGATATCGCCAGCGTCGCACAGCATGTTTGCTTGTCGCCGTCGCGTCTGTCACATCTTTTCCGCCAGCAGTTAGGGATTAGCGTCTTAAGCTGGCGCGAGGACCAACGTATCAGCCAGGCGAAGCTGCTTTTGAGCACCACCCGGATGCCTATCGCCACCGTCGGTCGCAATGTTGGTTTTGACGATCAACTCTATTTCTCGCGGGTATTTAAAAAATGCACCGGGGCCAGCCCGAGCGAGTTCCGTGCCGGTTTGGAAGAAAAAGTGAATGATGTAGCCGTCAAGTTGTCATGA |
| mKate | ATGTCAGAATTAATTAAAGAAAATATGCACATGAAATTATATATGGAAGGTACTGTCAACAATCATCATTTCAAATGCACATCCGAAGGTGAAGGTAAACCATATGAAGGCACACAAACAATGCGCATCAAAGCAGTTGAAGGTGGACCCCTGCCCTTTGCGTTTGACATTCTCGCAACGAGCTTTATGTACGGGTCTAAAACTTTTATCAATCACACCCAAGGCATTCCTGACTTTTTTAAACAGTCCTTTCCTGAAGGCTTTACCTGGGAACGTGTAACAACTTATGAAGATGGCGGTGTACTTACAGCAACTCAAGATACGAGTTTACAAGATGGCTGTCTGATTTACAATGTTAAAATCCGTGGCGTAAATTTCCCGAGTAACGGACCCGTAATGCAAAAAAAAACTCTTGGTTGGGAAGCATCAACAGAAACCTTATATCCTGCGGACGGTGGCTTAGAAGGACGCGCAGACATGGCACTGAAATTAGTTGGAGGCGGTCATTTAATCTGCAACCTGAAAACAACCTATCGTTCCAAAAAACCCGCTAAAAACCTTAAAATGCCTGGAGTATACTATGTTGATCGTCGCTTAGAACGTATTAAAGAAGCTGATAAAGAAACCTACGTTGAACAACATGAAGTAGCCGTAGCCCGTTATTGTGACCTTCCGTCGAAATTAGGACATCGTTGA |
| P_BAD_ | AGAAACCAATTGTCCATATTGCATCAGACATTGCCGTCACTGCGTCTTTTACTGGCTCTTCTCGCTAACCAAACCGGTAACCCCGCTTATTAAAAGCATTCTGTAACAAAGCGGGACCAAAGCCATGACAAAAACGCGTAACAAAAGTGTCTATAATCACGGCAGAAAAGTCCACATTGATTATTTGCACGGCGTCACACTTTGCTATGCCATAGCATTTTTATCCATAAGATTAGCGGATCCTACCTGACGCTTTTTATCGCAACTCTCTACTGTTTCTCCATACCCG |
| TetR | ATGTCCAGATTAGATAAAAGTAAAGTGATTAACAGCGCATTAGAGCTGCTTAATGAGGTCGGAATCGAAGGTTTAACAACCCGTAAACTCGCCCAGAAGCTAGGTGTAGAGCAGCCTACATTGTATTGGCATGTAAAAAATAAGCGGGCTTTGCTCGACGCCTTAGCCATTGAGATGTTAGATAGGCACCATACTCACTTTTGCCCTTTAGAAGGGGAAAGCTGGCAAGATTTTTTACGTAATAACGCTAAAAGTTTTAGATGTGCTTTACTAAGTCATCGCGATGGAGCAAAAGTACATTTAGGTACACGGCCTACAGAAAAACAGTATGAAACTCTCGAAAATCAATTAGCCTTTTTATGCCAACAAGGTTTTTCACTAGAGAATGCATTATATGCACTCAGCGCTGTGGGGCATTTTACTTTAGGTTGCGTATTGGAAGATCAAGAGCATCAAGTCGCTAAAGAAGAAAGGGAAACACCTACTACTGATAGTATGCCGCCATTATTACGACAAGCTATCGAATTATTTGATCACCAAGGTGCAGAGCCAGCCTTCTTATTCGGCCTTGAATTGATCATATGCGGATTAGAAAAACAACTTAAATGTGAAAGTGGGTCCTGA |
| sfGFP | ATGCGTAAAGGCGAAGAGCTGTTCACTGGTGTCGTCCCTATTCTGGTGGAACTGGATGGTGATGTCAACGGTCATAAGTTTTCCGTGCGTGGCGAGGGTGAAGGTGACGCAACTAATGGTAAACTGACGCTGAAGTTCATCTGTACTACTGGTAAACTGCCGGTACCTTGGCCGACTCTGGTAACGACGCTGACTTATGGTGTTCAGTGCTTTGCTCGTTATCCGGACCATATGAAGCAGCATGACTTCTTCAAGTCCGCCATGCCGGAAGGCTATGTGCAGGAACGCACGATTTCCTTTAAGGATGACGGCACGTACAAAACGCGTGCGGAAGTGAAATTTGAAGGCGATACTCTGGTAAACCGCATTGAGCTGAAAGGCATTGACTTTAAAGAAGACGGCAATATCCTGGGCCATAAGCTGGAATACAATTTTAACAGCCACAATGTTTACATCACCGCCGATAAACAAAAAAATGGCACTAAAGCGAATTTTAAAATTCGCCACAACGTGGAGGATGGCAGCGTGCAGCTGGCTGATCACTACCAGCAAAACACTCCAATCGGTGATGGTCCTGTTCTGCTGCCAGACAATCACTATCTGAGCACGCAAAGCGTTCTGTCTAAAGATCCGAACGAGAAACGCGATCATATGGTTCTGCTGGAGTTCGTAACCGCAGCGGGCATCACGCATGGTATGGATGAACTGTAC |
| pBBR1 | CTACCGGCGCGGCAGCGTTACCCGTGTCGGCGGCTCCAACGGCTCGCCATCGTCCAGAAAACACGGCTCATCGGGCATCGGCAGGCGCTGCTGCCCGCGCCGTTCCCATTCCTCCGTTTCGGTCAAGGCTGGCAGGTCTGGTTCCATGCCCGGAATGCCGGGCTGGCTGGGCGGCTCCTCGCCGGGGCCGGTCGGTAGTTGCTGCTCGCCCGGATACAGGGTCGGGATGCGGCGCAGGTCGCCATGCCCCAACAGCGATTCGTCCTGGTCGTCGTGATCAACCACCACGGCGGCACTGAACACCGACAGGCGCAACTGGTCGCGGGGCTGGCCCCACGCCACGCGGTCATTGACCACGTAGGCCGACACGGTGCCGGGGCCGTTGAGCTTCACGACGGAGATCCAGCGCTCGGCCACCAAGTCCTTGACTGCGTATTGGACCGTCCGCAAAGAACGTCCGATGAGCTTGGAAAGTGTCTTCTGGCTGACCACCACGGCGTTCTGGTGGCCCATCTGCGCCACGAGGTGATGCAGCAGCATTGCCGCCGTGGGTTTCCTCGCAATAAGCCCGGCCCACGCCTCATGCGCTTTGCGTTCCGTTTGCACCCAGTGACCGGGCTTGTTCTTGGCTTGAATGCCGATTTCTCTGGACTGCGTGGCCATGCTTATCTCCATGCGGTAGGGGTGCCGCACGGTTGCGGCACCATGCGCAATCAGCTGCAACTTTTCGGCAGCGCGACAACAATTATGCGTTGCGTAAAAGTGGCAGTCAATTACAGATTTTCTTTAACCTACGCAATGAGCTATTGCGGGGGGTGCCGCAATGAGCTGTTGCGTACCCCCCTTTTTTAAGTTGTTGATTTTTAAGTCTTTCGCATTTCGCCCTATATCTAGTTCTTTGGTGCCCAAAGAAGGGCACCCCTGCGGGGTTCCCCCACGCCTTCGGCGCGGCTCCCCCTCCGGCAAAAAGTGGCCCCTCCGGGGCTTGTTGATCGACTGCGCGGCCTTCGGCCTTGCCCAAGGTGGCGCTGCCCCCTTGGAACCCCCGCACTCGCCGCCGTGAGGCTCGGGGGGCAGGCGGGCGGGCTTCGCCCTTCGACTGCCCCCACTCGCATAGGCTTGGGTCGTTCCAGGCGCGTCAAGGCCAAGCCGCTGCGCGGTCGCTGCGCGAGCCTTGACCCGCCTTCCACTTGGTGTCCAACCGGCAAGCGAAGCGCGCAGGCCGCAGGCCGGAGGCTTTTCCCCAGAGAAAATTAAAAAAATTGATGGGGCAAGGCCGCAGGCCGCGCAGTTGGAGCCGGTGGGTATGTGGTCGAAGGCTGGGTAGCCGGTGGGCAATCCCTGTGGTCAAGCTCGTGGGCAGGCGCAGCCTGTCCATCAGCTTGTCCAGCAGGGTTGTCCACGGGCCGAGCGAAGCGAGCCAGCCGGTGGCCGCTCGCGGCCATCGTCCACATATCCACGGGCTGGCAAGGGAGCGCAGCGACCGCGCAGGGCGAAGCCCGGAGAGCAAGCCCGTAGGGG |
